# Atypical p38 Signaling, Activation, and Implications for Disease

**DOI:** 10.3390/ijms22084183

**Published:** 2021-04-17

**Authors:** Jeremy C. Burton, William Antoniades, Jennifer Okalova, Morgan M. Roos, Neil J. Grimsey

**Affiliations:** Department of Pharmaceutical and Biomedical Sciences, College of Pharmacy, University of Georgia, Athens, GA 30602, USA; jb22540@uga.edu (J.C.B.); William.Antoniades@uga.edu (W.A.); Jennifer.Okalova@uga.edu (J.O.); mmr82114@uga.edu (M.M.R.)

**Keywords:** MAPK, p38, atypical signaling, vascular disease, GPCRs, kinases, mechanisms

## Abstract

The mitogen-activated protein kinase (MAPK) p38 is an essential family of kinases, regulating responses to environmental stress and inflammation. There is an ever-increasing plethora of physiological and pathophysiological conditions attributed to p38 activity, ranging from cell division and embryonic development to the control of a multitude of diseases including retinal, cardiovascular, and neurodegenerative diseases, diabetes, and cancer. Despite the decades of intense investigation, a viable therapeutic approach to disrupt p38 signaling remains elusive. A growing body of evidence supports the pathological significance of an understudied atypical p38 signaling pathway. Atypical p38 signaling is driven by a direct interaction between the adaptor protein TAB1 and p38α, driving p38 autophosphorylation independent from the classical MKK3 and MKK6 pathways. Unlike the classical MKK3/6 signaling pathway, atypical signaling is selective for just p38α, and at present has only been characterized during pathophysiological stimulation. Recent studies have linked atypical signaling to dermal and vascular inflammation, myocardial ischemia, cancer metastasis, diabetes, complications during pregnancy, and bacterial and viral infections. Additional studies are required to fully understand how, when, where, and why atypical p38 signaling is induced. Furthermore, the development of selective TAB1-p38 inhibitors represents an exciting new opportunity to selectively inhibit pathological p38 signaling in a wide array of diseases.

## 1. Introduction

**Figure 1:**
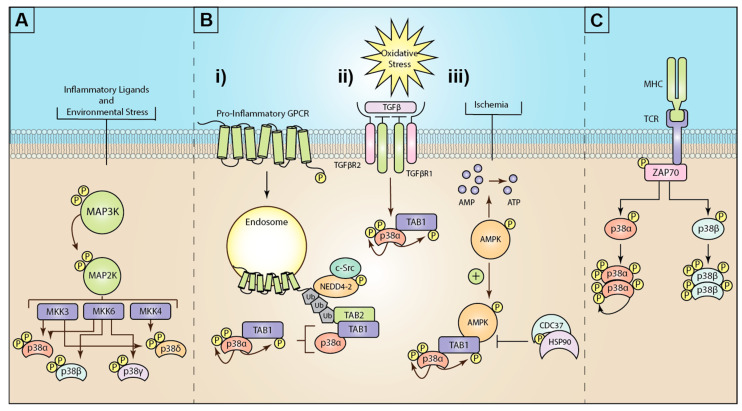
Mechanisms of mitogen-activated protein kinase (MAPK) p38 activation: (**A**) Inflammatory ligands and environmental stress trigger the activation of a three-tiered kinase cascade. Environmental or inflammatory ligands induce the activation of MAP3Ks through a complex array of different mechanisms. MAP3Ks then activate the critical MAP2Ks, MKK3, MKK6, or (less commonly) MKK4. These MAP2Ks can then differentially activate the four isoforms of p38 (α, β, γ, and δ). (**B**) The known mechanisms for atypical p38 signaling are (i) GPCR stimulation triggers G-protein dependent c-Src phospho-activation of the E3 ubiquitin ligase neural precursor cell expressed developmentally downregulated 4-2 (NEDD4-2). GPCRs recruit and are ubiquitinated by NEDD4-2. K63 ubiquitin chains recruit the ubiquitin-binding adaptor protein TAK1-binding protein 2 (TAB2). In turn, TAB2 then recruits TAB1, which binds and induces autophosphorylation of p38α. (ii) Oxidative stress triggers TGFβ activation, which drives TAB1 and p38 activation, although the exact mechanism is unclear. (iii) Ischemia or hypoxia events drive activation of AMP-activated protein kinase (AMPK), which in turn promotes the formation of the TAB1-p38α complex and p38α autophosphorylation. This process is negatively regulated by the heat shock protein 90 (HSP90)-Cdc37 complex. (**C**) T-cell receptor (TCR) ligation to major histocompatibility complex (MHC) drives intracellular activation of the src-family zeta-chain-associated protein kinase 70 (Zap70). Zap70 phosphorylates p38 at tyrosine 323, enabling autophosphorylation of p38α, or β.

The p38 mitogen-activated protein kinase (MAPK) family are critical cellular signaling regulators that drive many physiological and pathophysiological pathways. Therefore, it is not surprising that since their discovery in 1994 [1], over 45,000 research articles and reviews have been published describing the mechanism of p38 activation and the role of p38 during development and disease progression. The broader MAPK family includes c-Jun activated Kinase (JNK), extracellular signal-related kinase 1 and 2 (ERK1/2), and protein kinase B, also known as AKT kinase (AKT), all of which are critical in regulating a multitude of cellular processes from cell division to cell death and everything in between. Cellular stimuli/stress induces the activation of MAPKs, including hormones, growth factors, and cytokines, as well as environmental stressors such as osmotic shock, UV radiation, and ischemic injury [2]. As such, p38 MAPKs have been the subject of intense study to generate clinically effective therapeutics. Despite ongoing clinical trials for many diseases, including ischemic cardiac damage, COPD, multiple cancers, various neuropathies, and ARDS/COVID-19, only one non-selective p38 inhibitor (pirfenidone) has been approved for clinical use to treat idiopathic pulmonary fibrosis [3,4]. An underlying contributor to the loss of efficacy and on-target toxicity of these drugs is thought to be due to the ubiquitous and critical role p38 plays in normal physiology. Additionally, almost all current approaches have centered around therapeutics that target the ATP-binding site of p38 resulting in blockade of all p38 activity, both physiological and pathophysiological, regardless of the stimulus. Therefore, there is an increased focus on researching the downstream signaling targets of p38 induced only during disease progression, such as the critical inflammatory kinase MAPK-activated protein kinase 2 (MK2), or the alternative p38 activation pathways selectively induced during inflammation and disease progression (see Figure 1).

In light of the sheer volume of p38 research articles and the wealth of excellent reviews available, it would be impractical and redundant to cover all aspects of p38 MAPK signaling. Therefore, this review will initially provide a brief overview of the history of p38 and the many roles it plays in disease progression. This will be followed by a more focused examination of the novel atypical p38 activation pathways, specifically including atypical p38 activation by GPCRs, their implications for disease progression, and therapeutic intervention. In comparison to classical p38 activity, atypical p38 signaling has been understudied with only 44 publications, however, this growing body of work represents a fresh perspective on p38 activity and function in disease.

## 2. Classical Activation of Mitogen-Activated Protein Kinases (MAPK)

The classical pathway for MAPK activation is through a three-tiered kinase cascade, where MAP kinase kinase kinases activate a MAP kinase kinase which in turn activate MAPKs such as p38 (Figure 1). The most direct regulators of MAPK activity are the serine/threonine MAP2Ks that phosphorylate conserved threonine (Thr) and tyrosine (Tyr) sites on the activation loop of MAPKs [5]. Phosphorylation of the activation loop induces a conformational change to open the substrate-binding site [6]. One distinct feature of the subfamilies of MAPKs is their activating phosphorylation motifs. C-Jun N-terminal kinases (JNK) feature a Thr-Pro-Tyr sequence, extracellular-signal-regulated kinase (ERK) have a Thr-Glu-Tyr sequence, and p38 MAPK uses Thr-Gly-Tyr [7]. P38 MAPK was initially discovered as a MAP kinase activated in response to endotoxin with a sequence distinct from MAPK1 (ERK1) [1]. Further studies revealed p38 to be activated by a pair of unique MAP2Ks (MAPKK3/MKK3 and MAPKK6/MKK6) [6,8].

### 2.1. Activation of p38 by MAPKK3 and MAPKK6

MKK6 and MKK3 share a high degree of sequence homology with an 86% amino acid identity and selectively activate p38 MAPK over other MAP2Ks [7,9]. MKK3/6 are ubiquitously expressed in all tissues, although MKK3 and MKK6 have differing expression levels [10,11]. While MKK3/6 preferentially activate p38 MAPK, they can also activate other MAPK family members, such as JNK [12]. However, MKK3/6 are essential for classical p38 activation through phosphorylation of threonine [T180] and tyrosine (Y182) residues on the active loop of p38 [13]. Although under extreme conditions, p38 can also be activated by MKK4, typically selective to JNK [14]. The functional role of MKK3/6 is further emphasized through embryonic lethality seen in MKK3/6 double knockout mice (*mkk3^−/−^*, *mkk6^−/−^*), suggesting functional conservation [14], while recent evidence demonstrates that MKK3 and MKK6 can differentially activate specific p38 isoforms (see below) [15]. 

MAP2Ks are activated by MAP3Ks, which are less specific than MAP2Ks and activate an array of regulatory proteins. MAP3Ks are categorized into three broad families: MAPK/ERK kinase (MEK) kinases, mixed lineage kinases (MLKs), and thousand and one kinases (TAOs) [2]. Several factors regulate MAP3Ks, such as membrane recruitment, oligomerization, and phosphorylation [16]. Over 50 different MAP3Ks and adaptors can regulate MAP2K activation; many of the activation and recruitment mechanisms are still being actively investigated and substantial gaps remain in the pathways for activation. One example for adaptor-mediated activation is the MAP3K transforming growth factor-β-activated kinase (TAK1)-dependent MKK3/6 activation. TAK1 has a direct role in p38 MAPK activation as a mediator of the transforming growth factor-β signaling pathway [9,12], and several other common inflammatory ligands including IL-1β, TNFα, and LPS [17,18,19]. Critically, TAK1 is activated through direct binding to the adaptor proteins, TAK1-binding protein 1 and 2 (TAB1 and TAB2) [20]. In contrast to the MKK3/6-dependent pathway, recent studies have identified two atypical activation pathways, discussed below.

### 2.2. Distribution, Activation, and Function of the p38 Isoforms

There are four isoforms of p38 (α, β, γ, and δ). MAPK p38α is the founding member of the family and is ubiquitously expressed throughout the body. The four isoforms share a high degree of homology, p38β with 74% homology to p38α, p38γ with 63% homology to p38α, and p38δ with 60% homology to p38α [21,22,23]. Contrary to p38α, the other isoforms display differential tissue expression patterns. P38β is expressed mostly in the brain, heart, and lungs, p38γ is only expressed in skeletal muscle, and p38δ is expressed in the lungs and kidneys [13,21,22,23]. It is therefore not surprising that there is predicted to be little to no functional redundancy in their activity. However, MKK3 and MKK6 can differentially activate the separate isoforms, but all isoforms can be activated by MKK6 [15]. For example, MKK3/6 are both essential for activation of p38β and p38γ after environmental stress. While MKK6 regulates p38γ after TNFα stimulation, MKK3 activates p38δ after UV radiation, hyperosmotic anisomycin, and TNFα stimulation [15]. Furthermore, p38δ is activated by MKK4 more so than the other isoforms [24]. Even though p38α and p38β experience similar phosphorylation levels, activation of p38β is more often carried out by MKK6 [15,21]. Opposingly, MKK3 is demonstrated to be the primary activator of p38δ [15]. Whereas, p38γ, can be activated by MKK3 and MKK6 [15]. 

Notably, p38α is the only isoform that is essential for embryonic development, where it regulates placental vasculogenesis and morphogenesis [25,26]. Additionally, while some studies argue for it, p38β cannot compensate for p38α-controlled embryonic development, and it has instead been suggested that p38β is redundant when in the presence of a functional p38α [27,28].

The differential activation and signal transduction by MAPKs appear to be in part regulated by binding to specific scaffold proteins [29,30,31,32]. Scaffolding proteins residing in different subcellular locations may assist in the spatiotemporal activation of MAPKs. An example of which is osmotic stress that induces the formation of a complex, including Rac GTPase osmosensing scaffold for MEKK3 (OSM), MEKK3, and MKK3 for specific activation of p38 [33]. In comparison, the PB1 domain of MAPK kinase of ERK kinase (MEK2) drives endosomal ERK1/2 activation [34]. Furthermore, recent studies have shown that GPCR ubiquitination causes p38α activation through an atypical mechanism, utilizing TAB1 and TAB2 to form a signaling complex at endosomal structures to enhance vascular inflammation and endothelial barrier disruption [31].

### 2.3. P38 Substrate Activation

As the downstream signal transduction pathways for p38 are highly complex, we refer the reader to several outstanding and exhaustive reviews [35,36,37]. Briefly, the first downstream targets identified for p38 MAPK were the mitogen-activated protein kinase-activated protein kinase 2 and 3 (MAPKAPK2, MAPKAPK3, or MK2, MK3, respectively) [38,39]. Phosphorylated MK2 and MK3 can then further activate other substrates such as cyclic AMP-responsive element binding protein (CREB) [40] and heat shock protein 27 (HSP27) to regulate actin filament remodeling [41]. MK2 is also an important regulator of post-transcriptional regulation of gene expression through modulation of adenylate-uridylate-rich elements (ARE)-binding proteins tristetraprolin (TTP) and HuR (reviewed here [42]). However, mitogen- and stress-activated kinase 1 and 2 (MSK1 and MSK2) translocate to the nucleus to mediate activation of nucleosome components and transcription factors [43].

There are over 100 substrates identified for the p38 family with selective activation of specific substrates determined by the stimulation mechanism, including inflammation, DNA repair, cell differentiation, stem cell physiology, stress responses, and neuronal function [35,36,44]. An interesting problem in the field is determining how p38 can selectively modulate subsets of target proteins in different disease settings. One clue is that activation of p38 never occurs in isolation, with multiple signaling pathways working in synergy to regulate physiological outcomes. P38 substrate expression levels are often dynamically regulated and cross-talk between different signaling pathways are likely to contribute to the availability of specific substrates. Likewise, the magnitude of p38 activation, which is often robustly activated during disease is likely to influence which substrates can be phosphorylated and for how long. This raises the question of how p38 MAPK signaling can be turned off.

### 2.4. Signal Termination

With p38 MAPK playing a critical role in many cellular functions, dephosphorylation of both threonine and tyrosine residues in the active loop is required for inactivation of the kinase and signal termination. The most widely studied family responsible for dephosphorylating p38 is the dual specificity phosphatases (DUSPs) also referred to as MAPK phosphatases (MKPs). The DUSP family can dephosphorylate all members of the MAPK family. However, DUSP1/MKP1, DUSP10/MKP5, DUSP26/MKP8, and DUSP12 display a higher degree of specificity to p38α than the other DUSP family members [45,46,47], whereas no DUSPs have been reported for p38δ and p38γ. Recent studies have shown that temporal oscillations of MKP1 are key to robust proinflammatory gene expression [48]. Additional studies are required to determine whether the same phenomenon is displayed by all DUSP family members and whether MKP1 oscillations are required for all p38α activity. In addition to the DUSP family members, protein phosphatase 2 (PP2) [49], Wip1 [50], and calcyclin-binding protein/Siah-1 interacting protein (SIP1) [51] have all been shown to dephosphorylate p38. However, the broader roles of these phosphatases in p38 activity have yet to be established.

### 2.5. Molecular Inhibition

Since its discovery, p38 has been recognized as a potentially critical therapeutic target [52,53]. Multiple small molecule p38 kinase inhibitors have since been developed with tremendous specificity, largely owing to the rich structural information generated by X-ray crystallography studies available for the p38 family of kinases [54,55,56]. Many of these compounds have entered clinical trials, as shown in Table 1. These include inhibitors for the p38 kinase family (doramapimod, ralimetinib, and losmapimod) as well as more specific p38α inhibitors (PH-797804 and related pyridinone scaffold inhibitors). Pyridinone inhibitors exploit a unique binding model of a dual H-bond motif involving Met109 and Gly110 residues with a flipped backbone conformation of Gly110 in its apo state [57,58]. The unique methionine and glycine configuration in the gatekeeper region is only conserved in the human kinome in p38α/β and Myt-1, the latter of which bears little kinase resemblance to the former and has not shown to be cross-reactive with pyridinone scaffold inhibitors [59]. Specific p38 inhibitors almost invariably have been designed to target p38 kinase activity, primarily through binding to or near the ATP-binding pocket and display effectiveness at selectively inhibiting p38 in preclinical studies [35,57]. In early-stage investigations, many of these inhibitors show anti-inflammatory efficacy and favorable toxicity profiles [60,61,62,63,64], but so far none have achieved prolonged efficacy against chronic inflammatory disease, and only the p38γ inhibitor pirfenidone has reached the market for treatment of idiopathic pulmonary fibrosis. Many promising compounds have been reassigned for further investigation as combinatorial therapies such as repurposing ralimetinib for combination therapy in breast cancer (ClinicalTrials.gov ID: NCT01663857). Such an approach has proven effective for improving existing therapies, as seen in a study of doramapimod administration alongside antibiotics improving mycobacterium clearance in mice [65], and the well-studied losmapimod is currently being evaluated in a clinical trial for safety and efficacy to treat SARS-CoV-2 (ClinicalTrials.gov ID: NCT04511819).

The consistent short-lived efficacy of current inhibitors suggests that compensatory inflammatory pathways are upregulated over time in response to total p38 activity inhibition. While many well-designed investigations have studied p38 as a therapeutic target, Much remains unknown about p38 subcellular localization and what controls its access to downstream substrates after stimulation, especially pertaining to MKK3/6 verses atypical activation. Current investigations into inhibitor design are shifting away from targeting the catalytic site of p38 and instead focus on substrates and downstream signaling pathways [66,67,68,69,70,71]. Future therapeutics could avoid long-term efficacy issues from targeting the catalytic site by focusing on alternate druggable sites on p38. Several promising leads have recently been discovered. One example is the lead compound UM101, which binds to the glutamate-aspartate (ED) substrate-docking site rather than the catalytic domain. UM101 is selective for p38α and able to suppress LPS-induced acute lung injury in mice, inflammation, and endothelial barrier disruption in mice, while leaving anti-inflammatory MSK1 activation intact [67]. Another example targets a unique binding pocket in p38α, which is only bound by the adaptor protein TAB1 during atypical p38 activation. A virtual screen has revealed several promising lead compounds [66] and is described in the following section. However, these compounds have yet to be assessed in cell-based or animal models.

The burgeoning generation of selective atypical targets provides a promising new direction for clinically viable approaches for anti-p38 therapeutics. Furthermore, it is predicted that the combinatory therapies described above will provide a template moving forward to enable clinically viable strategies to target p38 activity.

## 3. Mechanisms of Atypical p38 Activation

MKK3/6 kinase activity is widely considered to be the primary mechanism for p38 phosphorylation. Nevertheless, there is a growing body of evidence to support alternative mechanisms for p38 activation (Figure 1). Two “atypical” or MKK3/6 independent mechanisms exist that facilitate activation of the p38α through autophosphorylation in cis, true autophosphorylation rather than phosphorylation of a neighboring p38 [72]. The first example of atypical p38 signaling was discovered in 2002, when p38α was shown to directly associate with transforming growth factor β-activated kinase 1 (TAK1) binding protein 1 (TAB1), an adaptor protein critical for both TGFβ and TAK1 signaling [73]. During osmotic stress responses [74], TAB1 is responsible for oligomerization and autophosphorylation of TAK1 after O-glycosylation, leading to TAK1 activation [75,76]. Conversely, in atypical p38 signaling, TAB1 binds directly and selectively to two discrete binding domains on p38α. Specifically, TAB1 residues 404–412 interact at a canonical site used by other p38 substrates, including MKK3 and MEF2a, and residues 389-394 bind to a non-canonical binding site on the c-terminal lobe of p38α. This site does not exist on any of the other p38 isoforms, and at the time of writing, no other proteins have been shown to bind to the same site on p38α [66,70]. The direct interaction of TAB1 with p38α induces a conformational change moving the active loop into the catalytic domain and enhancing ATP-binding, thus enabling cis-autophosphorylation of the active loop at Thr180 and Tyr182 [72]. Consequently, this leads to p38-induced phosphorylation of TAB1 at Ser423, downregulating TAB1 binding to TAK1 and inhibiting TAK1-mediated MKK3/6 activation [77]. Additional studies have also shown that TAB1 phosphorylation can alter its intracellular localization, where increased phosphorylation at S452/453/456/457 blocks its nuclear translocation causing TAB1 retention in the cytosol [78]. Intriguingly, TAB1 remains bound to p38α during atypical p38 activity, potentially suppressing the capacity of p38 nuclear translocation [70].

Reactive oxygen species are thought to be the initial driving force behind atypical p38 signaling in cardiac ischemia-reperfusion damage [70,72]. Similarly, cigarette smoke extract (CSE) induced oxidative stress in fetal tissue upregulating TGFβ production and resulting in TAB1-mediated p38 phosphorylation in a manner independent of TAK1 signaling or the ASK1-signalosome [79]. In a separate cardiac ischemia model, the TAB1-p38 interaction is upregulated in an AMPK-dependent manner [80] (Figure 1B ii). The interaction is negatively regulated by the HSP90/CDC37 chaperone complex in myocytes [81]. TAB1 expression is also negatively regulated by the E3 ligase itch through ubiquitin-mediated degradation. Where itch-deficient mice display dramatically increased dermal inflammation levels in an MKK3/6-independent manner [82]. The WW-domain in itch binds directly to a conserved PPXY motif in TAB1 (aa145–148). This interaction drives TAB1 K^48^-linked ubiquitination to regulate TAB1 turnover/degradation. TAB1 expression is significantly elevated in the absence of itch, leading to enhanced atypical p38 activation and increased cytokine production, including interleukin-6 (IL-6), interleukin-1beta (IL-1β), interleukin-11 (IL-11), and interleukin-19 (IL-19). Critically, Wang et al. in 2013 developed a peptide inhibitor fused to the HIV-TAT peptide, generating a cell-penetrating peptide inhibitor that selectively disrupts the TAB1 interaction with p38, substantially attenuating atypical p38 activation [83,84]. When used in the itch^−/−^ mice, the peptide blocked atypical p38α signaling and dermal inflammation was significantly suppressed [82]. Further studies have shown that mutation of a critical proline proximal to the p38 binding peptide of TAB1 (P419) blocks TAB1 binding to p38α and prevents atypical p38α signaling [31,72,85], as does mutation of four key residues within the p38α-binding peptide of TAB1 (V390A, Y392A, V408G, and M409A) [70,72]. Critically, unlike the systemic knockout of TAB1 or p38α, which are embryonically lethal [25,86], the TAB1 knock-in (TAB1-KI) mouse displays no physiological abnormalities but is protected from myocardial ischemic damage [70].

This critical interaction provides a novel opportunity to further develop the peptide inhibitors or screen for small molecule inhibitors to target atypical p38 signaling selectively. Indeed, using a virtual small fragment screen, a group of functionalized adamantanes, specifically 3-amino-1-adamantanol, was found to bind to a critical hydrophobic pocket, forming hydrogen bonds with two key residues, leucine 222 and 234, in the non-canonical TAB1 binding site on p38α. Further screening found there to be three distinct fragment binding sites within the non-canonical binding site. Linking sulfonamide scaffolds to the adamantanol generated a small molecule with a high affinity to the three regions in the non-canonical binding site [66]. Additional development of these compounds will hopefully yield a viable therapeutic. However, it remains to be shown whether these lead hits can block atypical p38 signaling in cells or in vivo.

Despite these detailed studies describing the exact molecular mechanism of TAB1-p38α interaction and degradation, there are significant gaps in our understanding, specifically for how osmotic stress, oxidative stress, LPS, or inflammatory cytokines such as TNF-α and IL-1β initiate the TAB1-p38α interaction and atypical p38 signal transduction. Conversely, recent studies have shown that a family of G protein-coupled receptors (GPCRs) can initiate the TAB1-p38 interaction through a novel ubiquitin-driven pathway (described below and Figure 1B). This is the first example of a clearly defined mechanism for the induction of atypical p38 signaling and demonstrates conservation of the mechanism for at least four GPCRs critical for vascular inflammatory signaling and vascular homeostasis [31,32,87].

In addition to TAB1, a second discrete mechanism for p38 autophosphorylation has also been demonstrated through src-family zeta-chain-associated protein kinase 70 (Zap70). This pathway is critical for T-cell activation through a T-cell receptor (TCR) specific mechanism [88]. In contrast to TAB1-mediated autophosphorylation, p38α and p38β isoforms are phosphorylated at Tyr323 by ZAP70, leading to dimerization and mutual trans-autophosphorylation of the kinases at Thr180 alone. Tyr323 is located on the L16 loop of p38, facilitating this autophosphorylation by inducing a shift in the flexible phosphorylation lip of p38 (residues 171–183) [89]. Together, both TAB1- and ZAP70-mediated autophosphorylation of p38 reveal the kinase’s atypical activation in an MKK3/6-independent manner. The functional significance of these distinct activation mechanisms is still unclear. Additional studies are required to elucidate how atypical activation alters p38α substrate activation and induction of distinct signal transduction events. Notably, p38α is phosphorylated at the same sites in both classical MKK3/6-mediated and TAB1-mediated signaling, indicating that differential downstream signaling may instead be regulated in a spatiotemporal context rather than kinase functionality.

## 4. Activation of Atypical p38 by GPCRs

As the most extensive and versatile family of membrane proteins, G protein-coupled receptors (GPCRs) regulate many cellular pathways by activating MAPKs via G protein-dependent and -independent mechanisms [90,91,92,93,94]. Many of the GPCR families can activate p38α, but until recently, the mechanism for GPCR-mediated p38α activation remained unclear or was predicted to be controlled through the classical MKK3/6 pathway. However, several recent studies have linked vascular inflammatory GPCRs to the activation of the TAB1-dependent atypical p38 signaling pathway [31,32,73,87,95,96]. The initial studies examined thrombin-mediated activation of the protease-activated receptor 1 (PAR1) in vascular endothelial cells. The authors noted that after activation, PAR1 was ubiquitinated, despite being trafficked and degraded in a ubiquitin-independent manner [95,97,98,99]. α-Thrombin, activation of PAR1 induces the receptor to couple to the G protein subunits Gα_q_ or Gα_12/13_ to induce activation of the proto-oncogene tyrosine-protein kinase c-Src (Src short for sarcoma) and subsequent activation of the E3 ubiquitin ligase, neural precursor cell expressed developmentally downregulated 4-2 (NEDD4-2) [32]. NEDD4-2 is one of a family of nine Homologous to E6-AP Carboxy Terminus (HECT) domain-containing E3 ligases and mediates the covalent coupling of ubiquitin to the intracellular c-tail or intracellular loops of GPCRs [31,87]. C-Src activates NEDD4-2 through tyrosine phosphorylation of a critical tyrosine residue, Y485, on a linker peptide between WW domain 2 and 3 (2,3 peptide). This 2, 3-linker peptide acts as a molecular switch that holds NEDD4-2 in an inactive conformation. Phosphorylation of Y485 by c-Src induces a conformational change that releases NEDD4-2 from an autoinhibited state. After activation, most likely at the plasma membrane, NEDD4-2 is recruited to PAR1, leading to PAR1 ubiquitination [32], although the exact mechanism as to how NEDD4-2 is recruited to PAR1 is unknown. Traditionally, GPCR ubiquitination serves as a sorting signal to cause endolysosomal trafficking and protein degradation [31,95]. However, in this case, NEDD4-2-mediated ubiquitination drives the recruitment of the TAB2-TAB1-p38 signaling complex [31,32,87,95]. TAB2 has an NP14 zinc finger (NZF) domain that binds to the lysine 63-linked NEDD4-2 ubiquitin chains and functions as an adaptor protein. It is predicted but has not been conclusively shown that TAB2 subsequently binds to and recruits TAB1 and p38α, inducing p38α autophosphorylation and TAB1 phosphorylation [31,100]. Interestingly, a structural homolog to TAB2, TAB3, is also able to bind to TAB1 to produce p38 pro-inflammatory signaling by GPCRs. However, it is not known what the contribution of each homolog is when expressed in the same cell or whether they are functionally redundant [87]. As stated above, the ubiquitinated endosomal receptors nucleate the formation and activation of the TAB1-p38α complex and increase TAB1 phosphorylation and stability [31]. It is still unclear whether GPCR-activated TAB1 sequesters p38 in the cytosol. Likewise, it is not known how TAB1-p38 signaling is terminated.

Importantly, this pathway is not unique just to PAR1 and α-thrombin. NEDD4-2 dependent regulation of atypical p38 signaling is also conserved for the purinergic receptor P2Y1. Furthermore, a recent study also demonstrated that the pathway is conserved for prostaglandin E2 (PGE2), histamine, ADP, and α-thrombin-mediated p38 activation and inflammatory cytokine production in primary human microvascular and macrovascular endothelial cells [87]. Additional studies are required to determine how many GPCRs utilize this pathway, whether atypical p38 signaling is critical for all cells, and how it selectively contributes to pathophysiological responses.

## 5. Pathophysiological Implications of MKK3/6-Dependent p38 MAPKs

As p38 MAPKs play a critical role in the modulation of many physiological processes, the dysregulation of their signaling pathways can result in the pathogenesis of a range of inflammatory diseases, neurological diseases, retinopathies, and cancers. There have been multiple recent outstanding studies and reviews that extensively cover the many pathological pathways controlled by classical p38 signaling, some examples are highlighted in Table 2.

Early studies revealed that p38 MAPKs have a central role in the development of various chronic inflammatory diseases due to pro-inflammatory cytokine (PIC) production [35,164]. Specifically, p38α MAPK signaling regulates the biosynthesis of many inflammatory mediators in cells of the immune system, epithelial cells, fibroblasts, and endothelial cells [165]. Excessive production of these mediators is associated with the pathological progression of acute and chronic inflammatory diseases including chronic obstructive pulmonary disease (COPD), rheumatoid arthritis (RA), gastritis, and psoriasis [35,166,167]. However, the story is complicated by a dichotomy of responses where p38 can exert both pro- and anti-inflammatory effect during disease progression. P38 can directly phosphorylate pro-inflammatory transcription factors such as MEF2C [168], and indirectly regulate inflammatory cytokine production through the MK2/3-TTP axis, where p38 phosphorylation of TTP prevents TTP-dependent degradation of AU-rich cytokine mRNA, leading to an accelerated inflammatory response [39,42,132]. As such p38 is an essential driver of inflammatory mediators such as COX2, MMP9, iNOS, TNFα, and IL6 [36,169,170,171,172]. Conversely, p38 also plays a central role in anti-inflammatory signaling. An example of this is p38-dependent regulation of IL10, a powerful anti-inflammatory cytokine which is important in resolving inflammatory insults [173,174]. IL10 expression is regulated through p38 activation of MSK1/2. Additionally, MSK1/2 also enhances DUSP1 expression which is required to restrain damaging hyperinflammation through dephosphorylation of p38 as described above [175].

Likewise, there is strong evidence for p38 in both tumor suppressive cellular homeostasis, balancing proliferation, differentiation, and apoptosis, and tumor promoting roles through promoting cell survival, proliferation, and angiogenesis [136]. Furthermore, p38 can both sensitize some tumor types to chemotherapy and facilitates resistance in others, where p38 inhibition may be beneficial in therapeutic approaches [136,176,177,178]. Of note, p38 MAPK activity and increased expression have been linked to the progression of breast cancer, prostate cancer, bladder cancer, liver cancer, lung cancer, thyroid cancers, leukemia, and many more [35,135,179,180]. In solid tumor biology, the p38 MAPK pathway has been shown to promote tumor cell survival and angiogenesis during periods of hypoxia, reoxygenation, and nutrient deficiency by inducing expression of metalloproteinases and vascular endothelial growth factor A (VEGFA) [135]. The context-dependent functions of p38 are, therefore, critical to determine the therapeutic potential of p38 inhibitors in cancer treatment, although p38 therapeutics have so far been unsuccessful in clinical trials.

In a similar manner, MAPK p38-induced cytokine expression during neuroinflammation accelerates the development of chronic neurodegenerative diseases such as multiple sclerosis (MS) [181], Alzheimer’s disease (AD) [144], and Parkinson’s disease (PD) [148], potentially through dysregulation of the neurovascular unit. During the pathophysiological progression of AD, elevated p38α MAPK signal transduction in both microglia and astrocytes results in subsequent neuroinflammation driving detrimental tau phosphorylation [145,146,148]. Conversely, p38γ signaling has recently been shown to mediate site-specific increases of post-synaptic tau phosphorylation and reduce tau-mediated memory deficits [147]. Furthermore, p38 MAPK-mediated microglial signaling is vital in dopamine neuron degeneration in PD patients [182]. Again, these data suggest that p38 therapeutics targeting the ATP pocket or catalytic domain are likely to be unsuccessful due to the dual roles of p38 in both physiological, protective, and pathological signaling.

## 6. Pathophysiological Implications of Atypical p38 Signaling

Contrary to the highly studied MKK3/6-dependent pathway, the impact of TAB1-p38-dependent signaling in physiology and disease remains largely understudied with just 44 research articles on the subject (Table 3). As mentioned above, the recent development of the viable p38α-KI mouse [108] or the TAB1-KI mouse [70] suggests that perturbation of the atypical pathway is less critical for developmental and physiological signaling compared to the embryonically lethal systemic knockout of p38α or TAB1 [25,86]. It is perhaps then not surprising that atypical p38 activation has so far only been identified as a contributor to disease progression, which will be discussed below.

There is a growing awareness that atypical p38α activation plays a key role multiple p38 driven pathologies. The initial studies describing atypical p38α activation demonstrate its role in ischemic cardiac damage, ischemia-reperfusion injury, and amyloidosis. In an MKK3^−/−^ ischemic mouse, the TAB1-p38 interaction was a leading contributor to necrosis in cardiomyocytes [105]. The role of atypical p38 was further confirmed in the progression of ischemic damage when a cell-penetrating inhibitor peptide was developed that reduced infarct size in ischemic rats [83]. Supporting this, the recent TAB1-KI mice where TAB1-induced autophosphorylation of p38 was genetically perturbed had significantly reduced infarction volume after induction of myocardial ischemia. Furthermore, the transphosphorylation of TAB1 was disabled [70], and cyclic GMP kinase 1 was found to inhibit TAB1-p38α to prevent apoptosis in cardiomyocytes during IR [104]. Additionally, basal activation of p38 autophosphorylation is suppressed by the HSP90/CDC37 complex where CD37 directly interacts with p38α [81]. Inhibition of HSP90 during cardiac stress is thought to dissociate HSP90 from p38α, enabling TAB1 interaction and p38α autophosphorylation to drive IL-6 and TNFα expression and cardiomyocyte apoptosis [81]. Additional studies have also shown that in a zebrafish model of amyloid light-chain (AL-LC) amyloidosis, AL-LC drives TAB1-p38α signaling causing cardiotoxic signaling, impaired cardiac function, pericardial edema, cell death, and subsequent heart failure [184,185].

Aside from the heart, p38 autophosphorylation has also been indicated in pathological inflammation in dermal disorders, preterm birth, and more broadly in vascular inflammation. In the itch^−/−^ mice, TAB1 expression is significantly enhanced, leading to robust p38 autophosphorylation and subsequent increases in inflammatory cytokine expression, immune cell recruitment, and spontaneous skin lesions [82]. The use of the cell-penetrating peptide inhibitor significantly reduced these phenotypes, suggesting that itch-mediated p38 signaling could be exploited therapeutically [82]. In the field of reproductive biology, term and preterm parturition are tied to oxidative-stress and inflammatory TGF-β-induced TAB1-p38 activity resulting in amniochorion senescence [79]. Atypical p38 is also considered an essential component of the careful balance of endothelial mesenchymal transition (EndoMT) and mesenchymal endothelial transition (MEndoT) in human and murine amnion cells that contributes to the timing of parturition [199].

Vascular inflammation also directly activates GPCR-dependent p38 signaling in endothelial cells. In these studies, GPCR ligand α-thrombin induces endothelial barrier disruption driving vascular leakage and permeability. Additionally, recent studies of GPCR-mediated TAB1-p38 activity have demonstrated that it is conserved in multiple endothelial vascular beds and activated by a family of GPCR ligands associated with inflammation such as histamine, PGE2, ADP, and potentially many others [31,32,87]. While it has not yet been definitively shown, it stands to reason that any cell that expresses these GPCR receptors has the potential to induce atypical p38 signaling. This being the case, it will be essential to understand the role of GPCR signaling in fibroblasts, epithelial cells, mural/pericyte cells, and neuronal cells. Therefore, the impact of GPCR-induced atypical signaling is likely to play an, as of yet, undiscovered or overlooked role in many other vascular inflammatory diseases.

Beyond the vasculature, the role of atypical p38 is also explored in the modulation of the immune system by inflammatory ligands, attenuation of the TCR, and response to pathogens. Basophils and eosinophils isolated from healthy patients undergo p38 autophosphorylation in response to cytokine exposure from TNFα and GM-CSF, contributing to prolonged inflammation like that seen in pulmonary inflammatory disorders [196]. Conversely, TAB1-p38 interaction is also associated with maintaining anergic CD4^+^ T-cells through increased expression of TAB1 following antigen exposure and abrogating TCR [195]. Similarly, TAB1-p38 drives T-cell senescence via an AMPK-dependent regulatory pathway, resulting in downregulation of TCR signalosome [197]. AMPK also plays an essential role in the TAB1-p38 activation of HSP27 in simulated sepsis, maintaining vascular integrity [191]. Intracellular infection leading to TAB1-p38 activity was first shown in macrophages in mice infected with *Toxoplasma gondii,* resulting in pro-inflammatory IL-12 production specific to atypical signaling [189]. *Leishmania* infection results in parasite GP63-induced degradation of TAB1 to reduce p38 activation [190], the reversal of which sharply attenuates infection [188]. These studies suggest a vital role for the TAB1-p38 interaction in the host defense during intracellular pathogen infection.

Another example of atypical p38 activation comes from a recent study that demonstrated that multiple viruses utilize atypical p38 signaling to drive viral infections. Inhibition of TAB1-dependent p38 activation impaired hepatitis C virus (HCV) assembly and viral replication. This was also confirmed for severe fever with thrombocytopenia syndrome virus (SFTSV), herpes simplex virus type 1 (HSV-1), and severe acute respiratory syndrome coronavirus 2 (SARS-CoV-2) [127]. Indeed, the p38 inhibitor losmapimod is currently in a clinical trial to treat SARS-CoV-2 (ClinicalTrials.gov ID: NCT04511819). It will be important for future studies to understand how atypical p38 signaling contributes to viral and bacterial infections and whether selective atypical p38 inhibitors could support current therapeutic regimens.

In the realm of type 1 diabetes, a link was found for TAB1-p38 interaction in the apoptosis of beta cells via oxidative stress by NO [193] and cytokine-induced beta-cell death [194]. These investigators noted that the effect of TAB1 signaling was specific to the TAB1α splicing product of the TAB1 gene located on chromosome 22, which has also been linked to systemic sclerosis and type 2 diabetes, hinting at a potential genetic component involving TAB1 mutation in the initiation of these diseases.

Contrary to TAB1-dependent signaling, Zap70-dependent activation of p38 is exclusive to T-cell activation via the TCR response, which is negatively regulated by p38 phosphorylation of upstream Zap70 [88,89]. However, a recent study also showed that TCR-mediated p38 activation occurs simultaneously through a classical kinase cascade and inflammatory augmentation by the alternative, atypical p38 activation. Intriguingly, it is suggested that uncoupling of the classical p38 activation mediated by the adaptor protein LAT and the guanine nuclear exchange factor, Son of Sevenless 1/2 (SOS1/2), reduced T-cell development and exacerbated autoimmune disease in mice [210]. At the same time, the genetic blockade of the TAB1-Zap70 suppressed T helper cell activation (T_H_1 and T_H_17) and expression of IFNγ and IL17. Indicating that both the classical and atypical p38 activation pathways could work synergistically to induce a balance between pro- and anti-inflammatory responses [210]. It is currently unclear whether there are some cases when TAB1-p38 activation may work in consort with MKK3/6, albeit in a TAK1-independent manner as TAB1 phosphorylation by p38 during atypical p38 signaling blocks TAB1′s interaction with TAK1 preventing TAB1-TAK1 dependent MKK3/6 activation [77].

## 7. Conclusions

The 25-year history of p38 MAPK has clearly demonstrated that this family of inflammatory kinases are essential for normal physiological processes and, if dysregulated, can be significant contributors to many diseases. Yet, despite many outstanding studies and carefully controlled clinical studies, therapeutic interventions targeting the conserved ATP pocket or structural scaffolds have so far been unsuccessful in the clinic. However, there are some promising avenues like targeting downstream signaling transducers such as MK2. Furthermore, the selective inhibition of pathological atypical p38 signaling represents a significantly under-investigated avenue and potentially critical target for therapeutic intervention.

Although there has been important progress in understanding the structural basis of the TAB1-p38 interaction and a clear mechanism has been defined for GPCR induced activation of atypical p38 signaling, there remain many gaps in our understanding of where, when, and why this pathway exists. There is still little understanding of how atypical p38 signaling alters the functional outcome of p38 activation to drive disease progression.

As outlined above, there is a growing body of clear evidence describing TAB1-dependent atypical p38 signaling (Table 3). Atypical p38 signaling has yet to be implemented in physiological pathways but is instead initiated only during disease progression, including cancer, viral infections, cardiac diseases, dermal inflammation, and vascular inflammation. This does raise a question of what evolutionary pressure resulted in the establishment of this pathway separate to MKK3/6 driven p38 activity. As more selective therapeutics are developed, it will be critical to determine whether blockade of TAB1-mediated p38 activation alters physiological or protective pathways. An important area of research should be in defining how TAB1 biases p38 signaling and identifying what substrates lay downstream of TAB1-p38. These studies would provide critical insight into how TAB1-p38 activity drives functional outcomes that, at present, appear to be only activated to drive disease progression.

Based on the significant role of GPCR ligands and p38 in the progression of so many diseases, it is clear that the current research has only just scratched the surface of the potential import of atypical p38 signaling. Future studies will yield critical detail to the broader mechanism of activation, and the development of TAB1-p38-selective inhibitors could pave the way forward to developing a clinically viable therapeutic.

## Figures and Tables

**Table 1:** Clinical trials targeting p38 mitogen-activated protein kinase (MAPK).

Compound	Isoform Specificity	Diseases Targeted	Identifier
AZD7624	p8α, p38β	Endotoxin-induced inflammation, COPD	NCT01937338 NCT02238483
LY2228820 (Ralimetinib)	p38 pan-inhibition	Ovarian cancer, glioblastoma (both concomitant), metastatic breast cancer	NCT02322853 NCT02364206 NCT01663857 NCT01393990
LY3007113	p38 pan-inhibition	Metastatic cancer	NCT01463631
VX-745 (Neflamapimod)	p38α	Alzheimer’s disease, Huntington disease, Lewy body dementia	NCT03980938 NCT04001517 NCT03402659 NCT03435861
VX-702	p38α	Rheumatoid arthritis	NCT00395577 NCT00205478
PH-797804	p38α	Rheumatoid arthritis, COPD	NCT01321463 NCT00559910 NCT01589614
SB681323 (Dilmapimod)	p38α	Neuropathic pain, COPD, ALI/ARDS, Coronary heart disease	NCT00134693 NCT00564746 NCT00390845 NCT00144859 NCT00320450 NCT00996840 NCT00291902
Losmapimod GW856553X or GSK-AHAB (Losmapimod)	p38 pan-inhibition	Acute coronary syndrome, COPD, neuropathic pain, SARS-CoV-2, atherosclerosis, acute coronary syndrome, focal segmental glomerulosclerosis, facioscapulohumeral muscular dystrophy	NCT04264442 NCT04511819 NCT02000440 NCT02299375 NCT04003974 NCT01541852 NCT01756495 NCT02145468 NCT01218126 NCT00633022
BMS-582949	p38 pan-inhibition	Arterial inflammation, atherosclerosis	NCT00162292 NCT00399906
ARRY-371797	p38α	LMNA-related dilated cardiomyopathy, rheumatoid arthritis, osteoarthritis of the knee, ankylosing spondylitis	NCT02351856 NCT03439514 NCT00729209 NCT01366014 NCT00811499
PF-03715455	p38α	Asthma, COPD	NCT02219048 NCT02366637
BIRB 796 (Doramapimod)	p38 pan-inhibition	Crohn’s disease, plaque-type psoriasis, rheumatoid arthritis, endotoxin-induced inflammation	NCT02214888 NCT02209753 NCT02209792 NCT02209779 NCT02211170
SCIO-469 (Talapimod)	p38α	Rheumatoid arthritis, multiple myeloma	NCT00095680 NCT00087867 NCT00043732 NCT00508768
Pirfenidone	p38γ	Idiopathic pulmonary fibrosis	NCT03208933
BCT-197 (Acumapimod)	p38α	COPD	NCT01332097 NCT02700919

**Table 2:** Pathological role of p38 MAPK signal transduction in a variety of diseases.

	Disease	Pathological Outcome	References
**Cardiovascular**	Myocardial infarction/Ischemia reperfusion	Induces overexpression of pro-inflammatory cytokines like IL-6, TNF-α, and IL-1β, and elevates intracellular calcium (Ca^2+^_i_) levels, inflammation, and apoptosis	[70,83,84,101,102,103,104,105,106,107,108]
	Diabetic cardiomyopathy	Overexpression of pro-inflammatory cytokines induces cardiomyocyte apoptosis	[109,110]
	Atherosclerosis	Promotes ANG-II-dependent MerTK shedding in macrophages resulting in defective efferocytosis and, in turn, induces plaque progression	[111,112,113,114,115]
**Pulmonary**	Chronic obstructive pulmonary disease (COPD)	Activates transcription factors and induces overexpression of pro-inflammatory cytokines and chemokines, amplifying lung inflammation	[116,117,118,119,120,121]
	Acute respiratory distress syndrome (ARDS)	Induces decreased corticosteroid responsiveness, alveolar macrophage-induced impairment of respiratory function, and overexpression of pro-inflammatory cytokines like IL-6, IL-8, TNF-α and IL-1β	[63,101,122,123]
	Acute lung injury (ALI)	Induces overexpression of pro-inflammatory cytokines like IL-6, TNF-α, and IL-1β, and cell apoptosis	[60,123,124,125,126]
	Viral infections and SARS-CoV-2	Induction of type 1 interferons, expression of IL-12, promotion of viral replication, expression of pro-inflammatory cytokines resulting in inflammation, thrombosis, and vasoconstriction in SARS-CoV-2	[60,127,128,129]
**Oncology**	Non-small cell lung cancer (NSCLC)	Enhances proliferation, migration, chemoresistance, and inflammatory cytokine expression	[130,131,132,133,134,135,136]
	Head and neck small cell carcinoma (HNSCC)	Inhibition of p38 increases HNSCC sensitivity to cisplatin, cannabinoids promote progressive HNSCC via p38 [42], increases mRNA stability via MK2, p38 isoforms as a diagnostic of HNSCC, and regulates angiogenesis and lymphangiogenesis	[137,138,139,140]
	Breast cancer	Elevated p38δ levels promote cell detachment, migration, invasion, and increased metastatic lesions, and inhibition of p38 triggers DNA damage and tumor cell death	[133,141,142]
	Bladder cancer	Induces cell invasion and metastasis by increasing MMP-2 and MMP-9 activity	[135,143]
**Neurodegenerative**	Alzheimer’s disease	Elevated p-p38 levels progress neuroinflammation tau phosphorylation, neurotoxicity, and synaptic dysfunction	[144,145,146,147]
	Parkinson’s disease	p-p38 overload induces a COX-2-mediated inflammation and subsequent dopaminergic neuron degeneration	[148,149,150]
	Amyotrophic lateral sclerosis (ALS)	Induces defects in axonal retrograde transport of signaling endosomes	[151,152,153]
	Spinal muscular atrophy	Induces p38 MAPK-dependent p53 phosphorylation leading to selective degeneration of motor neurons	[154]
**Ocular**	Age-related macular degeneration (AMD)	Induces VEGF expression and angiogenesis, regulates Ang-II-mediated MMP-2 and MMP-14, basigin expression, and extracellular matrix accumulation in AMD	[155,156]
	Diabetic retinopathy	ASK/p38 NLRP3 inflammasome signaling, retinal angiogenesis, retinal endothelial cell dysfunction, inner-blood-retinal-barrier leakage	[110,157,158,159,160,161]
	Glaucoma	Induces anterograde transport degradation and axon degeneration in the optic nerve	[162,163]

**Table 3:** Physiological roles of TAB1-dependent atypical p38 signaling.

Disease	Mechanism of p38 Autophosphorylation	Model	Specific Cell or Animal Line
Cardiovascular ischemia and reperfusion	TAB1-mediated	Murine in vivo [70,80,83,104,105]	MKK3^−/−^ [80,105]; C57BL/6 [80,104]; Sprague Dawley [80]; Wistar [83]; TAB1 KI [70]
		Murine in vitro [70,72,83,104,105,183]	H9c2 [105]; Sprague Dawley [83,104,183]; Wistar [83]; C57BL/6 [70,72]
		Human in vitro [70,83,84,104,108]	HEK293 [70,83,104,108]
		Structural modeling [66]	
Myocardial infarction, amyloidosis, and cardiomyopathy	TAB1-mediated	Murine in vivo [107]	Sprague Dawley
		Murine in vitro [81,107,184]	H9c2 [107]; Wistar [184]
		Human in vitro [184]	Patient heart
		Zebrafish in vivo [185]	
General inflammation and cancer	TAB1-mediated	Murine in vivo [31,82,186]	BALB/c [186]; CD1/CD1 [31]; C57BL/6, Itch^−/−^ [82]
		Murine in vitro [31,82,186]	Vβ8.1, OT-II [186]; TAB1^−/−^ [31]; C57BL/6, Itch^−/−^ [82]
		Human in vitro [31,32,87]	HUVEC [31,32,87]; HEK293 [31]; HDMEC [87]
		Structural modeling [89,187]	
Parasitic infection	TAB1-mediated	Murine in vivo [188]	BALB/c
		Murine in vitro [188,189,190]	RAW264.9 [188]; MKK3^−/−^ [189]; BALB/c [190]
Viral infection	TAB1-mediated	Murine in vitro [128]	C57BL/6, BC-1
		Human in vitro [127]	Huh7.5.1, HEK293, patient liver
Bacterial infection	TAB1-mediated	Human in vitro [191]	HPMEC
		Shrimp [192]	
Diabetes	TAB1-mediated	Murine in vitro [193,194]	β-TC6 [193,194]; Sprague Dawley, NMRI [194]
		Human in vitro [194]	Islet
Leukocyte dysfunction	TAB1-mediated	Murine in vivo [195]	Vβ8.1
		Murine in vitro [195]	2B4
		Human in vitro [196,197,198]	Patient blood
Pregnancy complications	TAB1-mediated	Murine in vitro [199]	CD-1
		Human in vitro [79,199]	Patient placenta
Other	TAB1-mediated	Murine in vitro [200,201]	MKK3^−/−^/6^−/−^ [200]; MKK3^−/−^ [201]
		Human in vitro [73,78,85,202]	HEK293 [73,78,85,202]; MDA231 [202]
		Structural modeling [203]	
Immune system (T-Cell) modulation	Zap70-mediated	Murine in vivo [88]	P116
		Murine in vitro [204,205,206,207]	Gadd45a^−/−^ [204]; CD4SP [205]; C57BL/6 [206,207]
		Human in vitro [88,208,209,210,211]	Jurkat, P116
		Chicken in vitro [210]	DT40

## Data Availability

No new data were created or analyzed in this study. Data sharing is not applicable to this article.

## References

[1] Han J., Lee J.D., Bibbs L., Ulevitch R.J. (1994). A MAP kinase targeted by endotoxin and hyperosmolarity in mammalian cells. Science.

[2] Kyriakis J.M., Avruch J. (2001). Mammalian mitogen-activated protein kinase signal transduction pathways activated by stress and inflammation. Physiol. Rev..

[3] King T.E., Bradford W.Z., Castro-Bernardini S., Fagan E.A., Glaspole I., Glassberg M.K., Gorina E., Hopkins P.M., Kardatzke D., Lancaster L. (2014). A phase 3 trial of pirfenidone in patients with idiopathic pulmonary fibrosis. N. Engl. J. Med..

[4] Valeyre D., Albera C., Bradford W.Z., Costabel U., King T.E., Leff J.A., Noble P.W., Sahn S.A., du Bois R.M. (2014). Comprehensive assessment of the long-term safety of pirfenidone in patients with idiopathic pulmonary fibrosis. Respirology.

[5] Min X., Akella R., He H., Humphreys J.M., Tsutakawa S.E., Lee S.J., Tainer J.A., Cobb M.H., Goldsmith E.J. (2009). The structure of the MAP2K MEK6 reveals an autoinhibitory dimer. Structure.

[6] Cuadrado A., Nebreda A.R. (2010). Mechanisms and functions of p38 MAPK signalling. Biochem. J..

[7] Raingeaud J., Whitmarsh A.J., Barrett T., Derijard B., Davis R.J. (1996). MKK3- and MKK6-regulated gene expression is mediated by the p38 mitogen-activated protein kinase signal transduction pathway. Mol. Cell. Biol..

[8] Derijard B., Raingeaud J., Barrett T., Wu I.H., Han J., Ulevitch R.J., Davis R.J. (1995). Independent human MAP-kinase signal transduction pathways defined by MEK and MKK isoforms. Science.

[9] Moriguchi T., Kuroyanagi N., Yamaguchi K., Gotoh Y., Irie K., Kano T., Shirakabe K., Muro Y., Shibuya H., Matsumoto K. (1996). A novel kinase cascade mediated by mitogen-activated protein kinase kinase 6 and MKK3. J. Biol. Chem..

[10] Tanaka N., Kamanaka M., Enslen H., Dong C., Wysk M., Davis R.J., Flavell R.A. (2002). Differential involvement of p38 mitogen-activated protein kinase kinases MKK3 and MKK6 in T-cell apoptosis. EMBO Rep..

[11] Lu H.T., Yang D.D., Wysk M., Gatti E., Mellman I., Davis R.J., Flavell R.A. (1999). Defective IL-12 production in mitogen-activated protein (MAP) kinase kinase 3 (Mkk3)-deficient mice. EMBO J..

[12] Lin A., Minden A., Martinetto H., Claret F.X., Lange-Carter C., Mercurio F., Johnson G.L., Karin M. (1995). Identification of a dual specificity kinase that activates the Jun kinases and p38-Mpk2. Science.

[13] Zhang Y.Y., Mei Z.Q., Wu J.W., Wang Z.X. (2008). Enzymatic activity and substrate specificity of mitogen-activated protein kinase p38alpha in different phosphorylation states. J. Biol. Chem..

[14] Brancho D., Tanaka N., Jaeschke A., Ventura J.J., Kelkar N., Tanaka Y., Kyuuma M., Takeshita T., Flavell R.A., Davis R.J. (2003). Mechanism of p38 MAP kinase activation in vivo. Genes Dev..

[15] Remy G., Risco A.M., Inesta-Vaquera F.A., Gonzalez-Teran B., Sabio G., Davis R.J., Cuenda A. (2010). Differential activation of p38MAPK isoforms by MKK6 and MKK3. Cell Signal..

[16] Marshall C.J. (1995). Specificity of receptor tyrosine kinase signaling: Transient versus sustained extracellular signal-regulated kinase activation. Cell.

[17] Irie T., Muta T., Takeshige K. (2000). TAK1 mediates an activation signal from toll-like receptor(s) to nuclear factor-kappaB in lipopolysaccharide-stimulated macrophages. FEBS Lett..

[18] Takaesu G., Surabhi R.M., Park K.J., Ninomiya-Tsuji J., Matsumoto K., Gaynor R.B. (2003). TAK1 is critical for IkappaB kinase-mediated activation of the NF-kappaB pathway. J. Mol. Biol..

[19] Brown K., Vial S.C., Dedi N., Long J.M., Dunster N.J., Cheetham G.M. (2005). Structural basis for the interaction of TAK1 kinase with its activating protein TAB1. J. Mol. Biol..

[20] Shim J.H., Xiao C., Paschal A.E., Bailey S.T., Rao P., Hayden M.S., Lee K.Y., Bussey C., Steckel M., Tanaka N. (2005). TAK1, but not TAB1 or TAB2, plays an essential role in multiple signaling pathways in vivo. Genes Dev..

[21] Jiang Y., Chen C., Li Z., Guo W., Gegner J.A., Lin S., Han J. (1996). Characterization of the structure and function of a new mitogen-activated protein kinase (p38beta). J. Biol. Chem..

[22] Jiang Y., Gram H., Zhao M., New L., Gu J., Feng L., Di Padova F., Ulevitch R.J., Han J. (1997). Characterization of the structure and function of the fourth member of p38 group mitogen-activated protein kinases, p38delta. J. Biol. Chem..

[23] Li Z., Jiang Y., Ulevitch R.J., Han J. (1996). The primary structure of p38 gamma: A new member of p38 group of MAP kinases. Biochem. Biophys. Res. Commun..

[24] O’Callaghan C., Fanning L.J., Barry O.P. (2014). p38δ MAPK: Emerging Roles of a Neglected Isoform. Int. J. Cell. Biol..

[25] Adams R.H., Porras A., Alonso G., Jones M., Vintersten K., Panelli S., Valladares A., Perez L., Klein R., Nebreda A.R. (2000). Essential role of p38alpha MAP kinase in placental but not embryonic cardiovascular development. Mol. Cell.

[26] Mudgett J.S., Ding J., Guh-Siesel L., Chartrain N.A., Yang L., Gopal S., Shen M.M. (2000). Essential role for p38alpha mitogen-activated protein kinase in placental angiogenesis. Proc. Natl. Acad. Sci. USA.

[27] Beardmore V.A., Hinton H.J., Eftychi C., Apostolaki M., Armaka M., Darragh J., McIlrath J., Carr J.M., Armit L.J., Clacher C. (2005). Generation and characterization of p38beta (MAPK11) gene-targeted mice. Mol. Cell. Biol..

[28] Greenblatt M.B., Shim J.H., Zou W., Sitara D., Schweitzer M., Hu D., Lotinun S., Sano Y., Baron R., Park J.M. (2010). The p38 MAPK pathway is essential for skeletogenesis and bone homeostasis in mice. J. Clin. Investig..

[29] Nakamura K., Johnson G.L. (2003). PB1 domains of MEKK2 and MEKK3 interact with the MEK5 PB1 domain for activation of the ERK5 pathway. J. Biol. Chem..

[30] Meister M., Tomasovic A., Banning A., Tikkanen R. (2013). Mitogen-Activated Protein (MAP) Kinase Scaffolding Proteins: A Recount. Int. J. Mol. Sci..

[31] Grimsey N.J., Aguilar B., Smith T.H., Le P., Soohoo A.L., Puthenveedu M.A., Nizet V., Trejo J. (2015). Ubiquitin plays an atypical role in GPCR-induced p38 MAP kinase activation on endosomes. J. Cell Biol..

[32] Grimsey N.J., Narala R., Rada C.C., Mehta S., Stephens B.S., Kufareva I., Lapek J., Gonzalez D.J., Handel T.M., Zhang J. (2018). A Tyrosine Switch on NEDD4-2 E3 Ligase Transmits GPCR Inflammatory Signaling. Cell Rep..

[33] Uhlik M.T., Abell A.N., Johnson N.L., Sun W., Cuevas B.D., Lobel-Rice K.E., Horne E.A., Dell’Acqua M.L., Johnson G.L. (2003). Rac-MEKK3-MKK3 scaffolding for p38 MAPK activation during hyperosmotic shock. Nat. Cell Biol..

[34] Galperin E., Sorkin A. (2008). Endosomal targeting of MEK2 requires RAF, MEK kinase activity and clathrin-dependent endocytosis. Traffic.

[35] Canovas B., Nebreda A.R. (2021). Diversity and versatility of p38 kinase signalling in health and disease. Nat. Rev. Mol. Cell Biol..

[36] Han J., Wu J., Silke J. (2020). An overview of mammalian p38 mitogen-activated protein kinases, central regulators of cell stress and receptor signaling. F1000Research.

[37] Cuenda A., Sanz-Ezquerro J.J. (2017). p38γ and p38δ: From Spectators to Key Physiological Players. Trends Biochem. Sci..

[38] Zu Y.L., Wu F., Gilchrist A., Ai Y., Labadia M.E., Huang C.K. (1994). The primary structure of a human MAP kinase activated protein kinase 2. Biochem. Biophys. Res. Commun..

[39] Soni S., Anand P., Padwad Y.S. (2019). MAPKAPK2: The master regulator of RNA-binding proteins modulates transcript stability and tumor progression. J. Exp. Clin. Cancer Res..

[40] Tan Y., Rouse J., Zhang A., Cariati S., Cohen P., Comb M.J. (1996). FGF and stress regulate CREB and ATF-1 via a pathway involving p38 MAP kinase and MAPKAP kinase-2. EMBO J..

[41] Guay J., Lambert H., Gingras-Breton G., Lavoie J.N., Huot J., Landry J. (1997). Regulation of actin filament dynamics by p38 map kinase-mediated phosphorylation of heat shock protein 27. J. Cell Sci..

[42] Soni S., Saroch M.K., Chander B., Tirpude N.V., Padwad Y.S. (2019). MAPKAPK2 plays a crucial role in the progression of head and neck squamous cell carcinoma by regulating transcript stability. J. Exp. Clin. Cancer Res..

[43] Reyskens K.M.S.E., Arthur J.S.C. (2016). Emerging Roles of the Mitogen and Stress Activated Kinases MSK1 and MSK2. Front. Cell Dev. Biol..

[44] Trempolec N., Dave-Coll N., Nebreda A.R. (2013). SnapShot: p38 MAPK substrates. Cell.

[45] Nunes-Xavier C., Romá-Mateo C., Ríos P., Tárrega C., Cejudo-Marín R., Tabernero L., Pulido R. (2011). Dual-specificity MAP kinase phosphatases as targets of cancer treatment. Anticancer Agents Med. Chem..

[46] Sun H., Charles C.H., Lau L.F., Tonks N.K. (1993). MKP-1 (3CH134), an immediate early gene product, is a dual specificity phosphatase that dephosphorylates MAP kinase in vivo. Cell.

[47] Chen H.F., Chuang H.C., Tan T.H. (2019). Regulation of Dual-Specificity Phosphatase (DUSP) Ubiquitination and Protein Stability. Int. J. Mol. Sci..

[48] Tomida T., Takekawa M., Saito H. (2015). Oscillation of p38 activity controls efficient pro-inflammatory gene expression. Nat. Commun..

[49] Takekawa M., Maeda T., Saito H. (1998). Protein phosphatase 2Calpha inhibits the human stress-responsive p38 and JNK MAPK pathways. EMBO J..

[50] Liu G., Hu X., Sun B., Yang T., Shi J., Zhang L., Zhao Y. (2013). Phosphatase Wip1 negatively regulates neutrophil development through p38 MAPK-STAT1. Blood.

[51] Topolska-Woś A.M., Rosińska S., Filipek A. (2017). MAP kinase p38 is a novel target of CacyBP/SIP phosphatase. Amino Acids.

[52] Kumar S., Boehm J., Lee J.C. (2003). p38 MAP kinases: Key signalling molecules as therapeutic targets for inflammatory diseases. Nat. Rev. Drug Discov..

[53] Adams J.L., Badger A.M., Kumar S., Lee J.C., King F.D., Oxford A.W. (2001). 1 p38 MAP Kinase: Molecular Target for the Inhibition of Pro-inflammatory Cytokines. Progress in Medicinal Chemistry.

[54] Xing L., Shieh H.S., Selness S.R., Devraj R.V., Walker J.K., Devadas B., Hope H.R., Compton R.P., Schindler J.F., Hirsch J.L. (2009). Structural bioinformatics-based prediction of exceptional selectivity of p38 MAP kinase inhibitor PH-797804. Biochemistry.

[55] Wrobleski S.T., Lin S., Dhar T.G., Dyckman A.J., Li T., Pitt S., Zhang R., Fan Y., Doweyko A.M., Tokarski J.S. (2013). The identification of novel p38α isoform selective kinase inhibitors having an unprecedented p38α binding mode. Bioorg. Med. Chem. Lett..

[56] Das J., Moquin R.V., Pitt S., Zhang R., Shen D.R., McIntyre K.W., Gillooly K., Doweyko A.M., Sack J.S., Zhang H. (2008). Pyrazolo-pyrimidines: A novel heterocyclic scaffold for potent and selective p38 alpha inhibitors. Bioorg. Med. Chem. Lett..

[57] Xing L. (2015). Clinical candidates of small molecule p38 MAPK inhibitors for inflammatory diseases. MAP Kinase.

[58] Devadas B., Selness S.R., Xing L., Madsen H.M., Marrufo L.D., Shieh H., Messing D.M., Yang J.Z., Morgan H.M., Anderson G.D. (2011). Substituted N-aryl-6-pyrimidinones: A new class of potent, selective, and orally active p38 MAP kinase inhibitors. Bioorg. Med. Chem. Lett..

[59] Selness S.R., Devraj R.V., Devadas B., Walker J.K., Boehm T.L., Durley R.C., Shieh H., Xing L., Rucker P.V., Jerome K.D. (2011). Discovery of PH-797804, a highly selective and potent inhibitor of p38 MAP kinase. Bioorg. Med. Chem. Lett..

[60] Grimes J.M., Grimes K.V. (2020). p38 MAPK inhibition: A promising therapeutic approach for COVID-19. J. Mol. Cell. Cardiol..

[61] Cheriyan J., Webb A.J., Sarov-Blat L., Elkhawad M., Wallace S.M., Mäki-Petäjä K.M., Collier D.J., Morgan J., Fang Z., Willette R.N. (2011). Inhibition of p38 mitogen-activated protein kinase improves nitric oxide-mediated vasodilatation and reduces inflammation in hypercholesterolemia. Circulation.

[62] Barbour A.M., Sarov-Blat L., Cai G., Fossler M.J., Sprecher D.L., Graggaber J., McGeoch A.T., Maison J., Cheriyan J. (2013). Safety, tolerability, pharmacokinetics and pharmacodynamics of losmapimod following a single intravenous or oral dose in healthy volunteers. Br. J. Clin. Pharmacol..

[63] Christie J.D., Vaslef S., Chang P.K., May A.K., Gunn S.R., Yang S., Hardes K., Kahl L., Powley W.M., Lipson D.A. (2015). A Randomized Dose-Escalation Study of the Safety and Anti-Inflammatory Activity of the p38 Mitogen-Activated Protein Kinase Inhibitor Dilmapimod in Severe Trauma Subjects at Risk for Acute Respiratory Distress Syndrome. Crit. Care Med..

[64] Strâmbu I.R., Kobalava Z.D., Magnusson B.P., MacKinnon A., Parkin J.M. (2019). Phase II Study of Single/Repeated Doses of Acumapimod (BCT197) to Treat Acute Exacerbations of COPD. J. Chronic Obstr. Pulm. Dis..

[65] Hölscher C., Gräb J., Hölscher A., Müller A.L., Schäfer S.C., Rybniker J. (2020). Chemical p38 MAP kinase inhibition constrains tissue inflammation and improves antibiotic activity in Mycobacterium tuberculosis-infected mice. Sci. Rep..

[66] Nichols C., Ng J., Keshu A., Kelly G., Conte M.R., Marber M.S., Fraternali F., De Nicola G.F. (2020). Mining the PDB for Tractable Cases Where X-ray Crystallography Combined with Fragment Screens Can Be Used to Systematically Design Protein-Protein Inhibitors: Two Test Cases Illustrated by IL1β-IL1R and p38α-TAB1 Complexes. J. Med. Chem..

[67] Shah N.G., Tulapurkar M.E., Ramarathnam A., Brophy A., Martinez R., Hom K., Hodges T., Samadani R., Singh I.S., MacKerell A.D. (2017). Novel Noncatalytic Substrate-Selective p38α-Specific MAPK Inhibitors with Endothelial-Stabilizing and Anti-Inflammatory Activity. J. Immunol..

[68] Yang L., Sun X., Ye Y., Lu Y., Zuo J., Liu W., Elcock A., Zhu S. (2019). p38α Mitogen-Activated Protein Kinase Is a Druggable Target in Pancreatic Adenocarcinoma. Front. Oncol..

[69] Wang C., Hockerman S., Jacobsen E.J., Alippe Y., Selness S.R., Hope H.R., Hirsch J.L., Mnich S.J., Saabye M.J., Hood W.F. (2018). Selective inhibition of the p38α MAPK-MK2 axis inhibits inflammatory cues including inflammasome priming signals. J. Exp. Med..

[70] De Nicola G.F., Bassi R., Nichols C., Fernandez-Caggiano M., Golforoush P.A., Thapa D., Anderson R., Martin E.D., Verma S., Kleinjung J. (2018). The TAB1-p38alpha complex aggravates myocardial injury and can be targeted by small molecules. JCI Insight.

[71] Astolfi A., Manfroni G., Cecchetti V., Barreca M.L. (2018). A Comprehensive Structural Overview of p38α Mitogen-Activated Protein Kinase in Complex with ATP-Site and Non-ATP-Site Binders. ChemMedChem.

[72] De Nicola G.F., Martin E.D., Chaikuad A., Bassi R., Clark J., Martino L., Verma S., Sicard P., Tata R., Atkinson R.A. (2013). Mechanism and consequence of the autoactivation of p38α mitogen-activated protein kinase promoted by TAB1. Nat. Struct. Mol. Biol..

[73] Ge B., Gram H., Di Padova F., Huang B., New L., Ulevitch R.J., Luo Y., Han J. (2002). MAPKK-independent activation of p38alpha mediated by TAB1-dependent autophosphorylation of p38alpha. Science.

[74] Inagaki M., Omori E., Kim J.Y., Komatsu Y., Scott G., Ray M.K., Yamada G., Matsumoto K., Mishina Y., Ninomiya-Tsuji J. (2008). TAK1-binding protein 1, TAB1, mediates osmotic stress-induced TAK1 activation but is dispensable for TAK1-mediated cytokine signaling. J. Biol. Chem..

[75] Scholz R., Sidler C.L., Thali R.F., Winssinger N., Cheung P.C., Neumann D. (2010). Autoactivation of transforming growth factor beta-activated kinase 1 is a sequential bimolecular process. J. Biol. Chem..

[76] Kishimoto K., Matsumoto K., Ninomiya-Tsuji J. (2000). TAK1 mitogen-activated protein kinase kinase kinase is activated by autophosphorylation within its activation loop. J. Biol. Chem..

[77] Cheung P.C., Campbell D.G., Nebreda A.R., Cohen P. (2003). Feedback control of the protein kinase TAK1 by SAPK2a/p38alpha. EMBO J..

[78] Wolf A., Beuerlein K., Eckart C., Weiser H., Dickkopf B., Muller H., Sakurai H., Kracht M. (2011). Identification and functional characterization of novel phosphorylation sites in TAK1-binding protein (TAB) 1. PLoS ONE.

[79] Richardson L., Dixon C.L., Aguilera-Aguirre L., Menon R. (2018). Oxidative stress-induced TGF-beta/TAB1-mediated p38MAPK activation in human amnion epithelial cells. Biol. Reprod..

[80] Li J., Miller E.J., Ninomiya-Tsuji J., Russell R.R., Young L.H. (2005). AMP-activated protein kinase activates p38 mitogen-activated protein kinase by increasing recruitment of p38 MAPK to TAB1 in the ischemic heart. Circ. Res..

[81] Ota A., Zhang J., Ping P., Han J., Wang Y. (2010). Specific regulation of noncanonical p38alpha activation by Hsp90-Cdc37 chaperone complex in cardiomyocyte. Circ. Res..

[82] Theivanthiran B., Kathania M., Zeng M., Anguiano E., Basrur V., Vandergriff T., Pascual V., Wei W.Z., Massoumi R., Venuprasad K. (2015). The E3 ubiquitin ligase Itch inhibits p38alpha signaling and skin inflammation through the ubiquitylation of Tab1. Sci. Signal..

[83] Wang Q., Feng J., Wang J., Zhang X., Zhang D., Zhu T., Wang W., Wang X., Jin J., Cao J. (2013). Disruption of TAB1/p38alpha interaction using a cell-permeable peptide limits myocardial ischemia/reperfusion injury. Mol. Ther..

[84] Pei Y.J., Wang Q.Y., Zhang J.Y., Guo Y.H., Feng J.N. (2018). Characterization and Evaluation of Key Sites in the Peptide Inhibitor of TAB1/p38 alpha Interaction. Int. J. Pept. Res. Ther..

[85] Zhou H., Zheng M., Chen J., Xie C., Kolatkar A.R., Zarubin T., Ye Z., Akella R., Lin S., Goldsmith E.J. (2006). Determinants that control the specific interactions between TAB1 and p38alpha. Mol. Cell. Biol..

[86] Komatsu Y., Shibuya H., Takeda N., Ninomiya-Tsuji J., Yasui T., Miyado K., Sekimoto T., Ueno N., Matsumoto K., Yamada G. (2002). Targeted disruption of the Tab1 gene causes embryonic lethality and defects in cardiovascular and lung morphogenesis. Mech. Dev..

[87] Grimsey N.J., Lin Y., Narala R., Rada C.C., Mejia-Pena H., Trejo J. (2019). G protein-coupled receptors activate p38 MAPK via a non-canonical TAB1-TAB2 and TAB1-TAB3 dependent pathway in endothelial cells. J. Biol. Chem..

[88] Salvador J.M., Mittelstadt P.R., Guszczynski T., Copeland T.D., Yamaguchi H., Appella E., Fornace A.J., Ashwell J.D. (2005). Alternative p38 activation pathway mediated by T cell receptor-proximal tyrosine kinases. Nat. Immunol..

[89] Diskin R., Lebendiker M., Engelberg D., Livnah O. (2007). Structures of p38alpha active mutants reveal conformational changes in L16 loop that induce autophosphorylation and activation. J. Mol. Biol..

[90] Flower D.R. (1999). Modelling G-protein-coupled receptors for drug design. Biochim. Biophys. Acta.

[91] Azzi M., Charest P.G., Angers S., Rousseau G., Kohout T., Bouvier M., Piñeyro G. (2003). β-Arrestin-mediated activation of MAPK by inverse agonists reveals distinct active conformations for G protein-coupled receptors. Proc. Natl. Acad. Sci. USA.

[92] Gutkind J.S. (2000). Regulation of Mitogen-Activated Protein Kinase Signaling Networks by G Protein-Coupled Receptors. Sci. STKE.

[93] McDonald P.H., Chow C.-W., Miller W.E., Laporte S.A., Field M.E., Lin F.-T., Davis R.J., Lefkowitz R.J. (2000). β-Arrestin 2: A Receptor-Regulated MAPK Scaffold for the Activation of JNK3. Science.

[94] Shenoy S.K., Lefkowitz R.J. (2005). Seven-transmembrane receptor signaling through beta-arrestin. Sci. STKE.

[95] Burton J.C., Grimsey N.J. (2019). Ubiquitination as a Key Regulator of Endosomal Signaling by GPCRs. Front. Cell Dev. Biol..

[96] Grimsey N.J., Trejo J. (2016). Integration of endothelial protease-activated receptor-1 inflammatory signaling by ubiquitin. Curr. Opin. Hematol..

[97] Dores M.R., Chen B., Lin H., Soh U.J., Paing M.M., Montagne W.A., Meerloo T., Trejo J. (2012). ALIX binds a YPX(3)L motif of the GPCR PAR1 and mediates ubiquitin-independent ESCRT-III/MVB sorting. J. Cell. Biol..

[98] Dores M.R., Grimsey N.J., Mendez F., Trejo J. (2016). ALIX Regulates the Ubiquitin-Independent Lysosomal Sorting of the P2Y1 Purinergic Receptor via a YPX3L Motif. PLoS ONE.

[99] Dores M.R., Lin H., Grimsey N.J., Mendez F., Trejo J. (2015). The alpha-arrestin ARRDC3 mediates ALIX ubiquitination and G protein-coupled receptor lysosomal sorting. Mol. Biol. Cell.

[100] Kulathu Y., Akutsu M., Bremm A., Hofmann K., Komander D. (2009). Two-sided ubiquitin binding explains specificity of the TAB2 NZF domain. Nat. Struct. Mol. Biol..

[101] Romero-Becerra R., Santamans A.M., Folgueira C., Sabio G. (2020). p38 MAPK pathway in the heart: New insights in health and disease. Int. J. Mol. Sci..

[102] Arabacilar P., Marber M. (2015). The case for inhibiting p38 mitogen-activated protein kinase in heart failure. Front. Pharmacol..

[103] Song N., Ma J., Meng X.W., Liu H., Wang H., Song S.Y., Chen Q.C., Liu H.Y., Zhang J., Peng K. (2020). Heat Shock Protein 70 Protects the Heart from Ischemia/Reperfusion Injury through Inhibition of p38 MAPK Signaling. Oxid. Med. Cell. Longev..

[104] Fiedler B., Feil R., Hofmann F., Willenbockel C., Drexler H., Smolenski A., Lohmann S.M., Wollert K.C. (2006). cGMP-dependent protein kinase type I inhibits TAB1-p38 mitogen-activated protein kinase apoptosis signaling in cardiac myocytes. J. Biol. Chem..

[105] Tanno M., Bassi R., Gorog D.A., Saurin A.T., Jiang J., Heads R.J., Martin J.L., Davis R.J., Flavell R.A., Marber M.S. (2003). Diverse mechanisms of myocardial p38 mitogen-activated protein kinase activation: Evidence for MKK-independent activation by a TAB1-associated mechanism contributing to injury during myocardial ischemia. Circ. Res..

[106] Zheng D.Y., Zhou M., Jin J., He M., Wang Y., Du J., Xiao X.Y., Li P.Y., Ye A.Z., Liu J. (2016). Inhibition of P38 MAPK Downregulates the Expression of IL-1beta to Protect Lung from Acute Injury in Intestinal Ischemia Reperfusion Rats. Mediators Inflamm..

[107] Du C.S., Yang R.F., Song S.W., Wang Y.P., Kang J.H., Zhang R., Su D.F., Xie X. (2010). Magnesium Lithospermate B Protects Cardiomyocytes from Ischemic Injury Via Inhibition of TAB1-p38 Apoptosis Signaling. Front. Pharmacol..

[108] Thapa D., Nichols C., Bassi R., Martin E.D., Verma S., Conte M.R., De Santis V., De Nicola G.F., Marber M.S. (2018). TAB1-Induced Autoactivation of p38α Mitogen-Activated Protein Kinase Is Crucially Dependent on Threonine 185. Mol. Cell. Biol..

[109] Wang S., Ding L., Ji H., Xu Z., Liu Q., Zheng Y. (2016). The Role of p38 MAPK in the Development of Diabetic Cardiomyopathy. Int. J. Mol. Sci..

[110] Al-Shabrawey M., Hussein K., Wang F., Wan M., Elmasry K., Elsherbiny N., Saleh H., Yu P.B., Tawfik A., Ibrahim A.S. (2020). Bone Morphogenetic Protein-2 Induces Non-Canonical Inflammatory and Oxidative Pathways in Human Retinal Endothelial Cells. Front. Immunol..

[111] Zhang Y., Wang Y., Zhou D., Zhang L.S., Deng F.X., Shu S., Wang L.J., Wu Y., Guo N., Zhou J. (2019). Angiotensin II deteriorates advanced atherosclerosis by promoting MerTK cleavage and impairing efferocytosis through the AT(1)R/ROS/p38 MAPK/ADAM17 pathway. Am. J. Physiol. Cell Physiol..

[112] Corre I., Paris F., Huot J. (2017). The p38 pathway, a major pleiotropic cascade that transduces stress and metastatic signals in endothelial cells. Oncotarget.

[113] Fisk M., Gajendragadkar P.R., Maki-Petaja K.M., Wilkinson I.B., Cheriyan J. (2014). Therapeutic potential of p38 MAP kinase inhibition in the management of cardiovascular disease. Am. J. Cardiovasc. Drugs.

[114] Reustle A., Torzewski M. (2018). Role of p38 MAPK in Atherosclerosis and Aortic Valve Sclerosis. Int. J. Mol. Sci..

[115] Seitz I., Hess S., Schulz H., Eckl R., Busch G., Montens H.P., Brandl R., Seidl S., Schomig A., Ott I. (2007). Membrane-type serine protease-1/matriptase induces interleukin-6 and -8 in endothelial cells by activation of protease-activated receptor-2: Potential implications in atherosclerosis. Arterioscler. Thromb. Vasc. Biol..

[116] Renda T., Baraldo S., Pelaia G., Bazzan E., Turato G., Papi A., Maestrelli P., Maselli R., Vatrella A., Fabbri L.M. (2008). Increased activation of p38 MAPK in COPD. Eur. Respir. J..

[117] Pelaia C., Vatrella A., Sciacqua A., Terracciano R., Pelaia G. (2020). Role of p38-mitogen-activated protein kinase in COPD: Pathobiological implications and therapeutic perspectives. Expert Rev. Respir. Med..

[118] Gaffey K., Reynolds S., Plumb J., Kaur M., Singh D. (2013). Increased phosphorylated p38 mitogen-activated protein kinase in COPD lungs. Eur. Respir. J..

[119] Armstrong J., Harbron C., Lea S., Booth G., Cadden P., Wreggett K.A., Singh D. (2011). Synergistic effects of p38 mitogen-activated protein kinase inhibition with a corticosteroid in alveolar macrophages from patients with chronic obstructive pulmonary disease. J. Pharmacol. Exp. Ther..

[120] Huang C., Xie M., He X., Gao H. (2013). Activity of sputum p38 MAPK is correlated with airway inflammation and reduced FEV1 in COPD patients. Med. Sci. Monit..

[121] Amano H., Murata K., Matsunaga H., Tanaka K., Yoshioka K., Kobayashi T., Ishida J., Fukamizu A., Sugiyama F., Sudo T. (2014). p38 Mitogen-activated protein kinase accelerates emphysema in mouse model of chronic obstructive pulmonary disease. J. Recept. Signal. Transduct..

[122] Feng Y., Fang Z., Liu B., Zheng X. (2019). p38MAPK plays a pivotal role in the development of acute respiratory distress syndrome. Clinics.

[123] Fang W., Cai S.X., Wang C.L., Sun X.X., Li K., Yan X.W., Sun Y.B., Sun X.Z., Gu C.K., Dai M.Y. (2017). Modulation of mitogenactivated protein kinase attenuates sepsisinduced acute lung injury in acute respiratory distress syndrome rats. Mol. Med. Rep..

[124] Bai X., Fan L., He T., Jia W., Yang L., Zhang J., Liu Y., Shi J., Su L., Hu D. (2015). SIRT1 protects rat lung tissue against severe burn-induced remote ALI by attenuating the apoptosis of PMVECs via p38 MAPK signaling. Sci. Rep..

[125] Xiong L.L., Tan Y., Ma H.Y., Dai P., Qin Y.X., Yang R.A., Xu Y.Y., Deng Z., Zhao W., Xia Q.J. (2016). Administration of SB239063, a potent p38 MAPK inhibitor, alleviates acute lung injury induced by intestinal ischemia reperfusion in rats associated with AQP4 downregulation. Int. Immunopharmacol..

[126] Li D., Ren W., Jiang Z., Zhu L. (2018). Regulation of the NLRP3 inflammasome and macrophage pyroptosis by the p38 MAPK signaling pathway in a mouse model of acute lung injury. Mol. Med. Rep..

[127] Cheng Y., Sun F., Wang L., Gao M., Xie Y., Sun Y., Liu H., Yuan Y., Yi W., Huang Z. (2020). Virus-induced p38 MAPK activation facilitates viral infection. Theranostics.

[128] Mikkelsen S.S., Jensen S.B., Chiliveru S., Melchjorsen J., Julkunen I., Gaestel M., Arthur J.S., Flavell R.A., Ghosh S., Paludan S.R. (2009). RIG-I-mediated activation of p38 MAPK is essential for viral induction of interferon and activation of dendritic cells: Dependence on TRAF2 and TAK1. J. Biol. Chem..

[129] Börgeling Y., Schmolke M., Viemann D., Nordhoff C., Roth J., Ludwig S. (2014). Inhibition of p38 mitogen-activated protein kinase impairs influenza virus-induced primary and secondary host gene responses and protects mice from lethal H5N1 infection. J. Biol. Chem..

[130] Pan J., Yang Q., Shao J., Zhang L., Ma J., Wang Y., Jiang B.H., Leng J., Bai X. (2016). Cyclooxygenase-2 induced β1-integrin expression in NSCLC and promoted cell invasion via the EP1/MAPK/E2F-1/FoxC2 signal pathway. Sci. Rep..

[131] Patel S., Vetale S., Teli P., Mistry R., Chiplunkar S. (2012). IL-10 production in non-small cell lung carcinoma patients is regulated by ERK, P38 and COX-2. J. Cell. Mol. Med..

[132] Singh R.K., Najmi A.K. (2019). Novel Therapeutic Potential of Mitogen-Activated Protein Kinase Activated Protein Kinase 2 (MK2) in Chronic Airway Inflammatory Disorders. Curr. Drug Targets.

[133] Wada M., Canals D., Adada M., Coant N., Salama M.F., Helke K.L., Arthur J.S., Shroyer K.R., Kitatani K., Obeid L.M. (2017). P38 delta MAPK promotes breast cancer progression and lung metastasis by enhancing cell proliferation and cell detachment. Oncogene.

[134] Zhu N., Zhang X.J., Zou H., Zhang Y.Y., Xia J.W., Zhang P., Zhang Y.Z., Li J., Dong L., Wumaier G. (2021). PTPL1 suppresses lung cancer cell migration via inhibiting TGF-β1-induced activation of p38 MAPK and Smad 2/3 pathways and EMT. Acta Pharmacol. Sin..

[135] Koul H.K., Pal M., Koul S. (2013). Role of p38 MAP Kinase Signal Transduction in Solid Tumors. Genes Cancer.

[136] Igea A., Nebreda A.R. (2015). The Stress Kinase p38alpha as a Target for Cancer Therapy. Cancer Res..

[137] Leelahavanichkul K., Amornphimoltham P., Molinolo A.A., Basile J.R., Koontongkaew S., Gutkind J.S. (2014). A role for p38 MAPK in head and neck cancer cell growth and tumor-induced angiogenesis and lymphangiogenesis. Mol. Oncol..

[138] Roy S., Roy S., Anuja K., Thakur S., Akhter Y., Padhi S., Banerjee B. (2021). p38 Mitogen-activated protein kinase modulates cisplatin resistance in Head and Neck Squamous Cell Carcinoma cells. Arch. Oral Biol..

[139] Liu C., Sadat S.H., Ebisumoto K., Sakai A., Panuganti B.A., Ren S., Goto Y., Haft S., Fukusumi T., Ando M. (2020). Cannabinoids Promote Progression of HPV-Positive Head and Neck Squamous Cell Carcinoma via p38 MAPK Activation. Clin. Cancer Res..

[140] Avendano M.S., Garcia-Redondo A.B., Zalba G., Gonzalez-Amor M., Aguado A., Martinez-Revelles S., Beltran L.M., Camacho M., Cachofeiro V., Alonso M.J. (2018). mPGES-1 (Microsomal Prostaglandin E Synthase-1) Mediates Vascular Dysfunction in Hypertension Through Oxidative Stress. Hypertension.

[141] Cánovas B., Igea A., Sartori A.A., Gomis R.R., Paull T.T., Isoda M., Pérez-Montoyo H., Serra V., González-Suárez E., Stracker T.H. (2018). Targeting p38α Increases DNA Damage, Chromosome Instability, and the Anti-tumoral Response to Taxanes in Breast Cancer Cells. Cancer Cell.

[142] Martínez-Limón A., Joaquin M., Caballero M., Posas F., de Nadal E. (2020). The p38 Pathway: From Biology to Cancer Therapy. Int. J. Mol. Sci..

[143] Kumar B., Koul S., Petersen J., Khandrika L., Hwa J.S., Meacham R.B., Wilson S., Koul H.K. (2010). p38 Mitogen-Activated Protein Kinase–Driven MAPKAPK2 Regulates Invasion of Bladder Cancer by Modulation of MMP-2 and MMP-9 Activity. Cancer Res..

[144] Lee J.K., Kim N.J. (2017). Recent Advances in the Inhibition of p38 MAPK as a Potential Strategy for the Treatment of Alzheimer’s Disease. Molecules.

[145] Corrêa S.A., Eales K.L. (2012). The Role of p38 MAPK and Its Substrates in Neuronal Plasticity and Neurodegenerative Disease. J. Signal. Transduct..

[146] Germann U.A., Alam J.J. (2020). P38α MAPK Signaling-A Robust Therapeutic Target for Rab5-Mediated Neurodegenerative Disease. Int. J. Mol. Sci..

[147] Ittner A., Asih P.R., Tan A.R.P., Prikas E., Bertz J., Stefanoska K., Lin Y., Volkerling A.M., Ke Y.D., Delerue F. (2020). Reduction of advanced tau-mediated memory deficits by the MAP kinase p38γ. Acta Neuropathol..

[148] He J., Zhong W., Zhang M., Zhang R., Hu W. (2018). P38 Mitogen-activated Protein Kinase and Parkinson’s Disease. Transl. Neurosci..

[149] Wang X., Sun X., Niu M., Zhang X., Wang J., Zhou C., Xie A. (2020). RAGE Silencing Ameliorates Neuroinflammation by Inhibition of p38-NF-κB Signaling Pathway in Mouse Model of Parkinson’s Disease. Front. Neurosci..

[150] Wang Q., Zheng H., Zhang Z.F., Zhang Y.X. (2008). Ginsenoside Rg1 modulates COX-2 expression in the substantia nigra of mice with MPTP-induced Parkinson disease through the P38 signaling pathway. Nan Fang Yi Ke Da Xue Xue Bao.

[151] De Vos K.J., Hafezparast M. (2017). Neurobiology of axonal transport defects in motor neuron diseases: Opportunities for translational research?. Neurobiol. Dis..

[152] Gibbs K.L., Greensmith L., Schiavo G. (2015). Regulation of Axonal Transport by Protein Kinases. Trends Biochem. Sci..

[153] Gibbs K.L., Kalmar B., Rhymes E.R., Fellows A.D., Ahmed M., Whiting P., Davies C.H., Greensmith L., Schiavo G. (2018). Inhibiting p38 MAPK alpha rescues axonal retrograde transport defects in a mouse model of ALS. Cell Death Dis..

[154] Simon C.M., Van Alstyne M., Lotti F., Bianchetti E., Tisdale S., Watterson D.M., Mentis G.Z., Pellizzoni L. (2019). Stasimon Contributes to the Loss of Sensory Synapses and Motor Neuron Death in a Mouse Model of Spinal Muscular Atrophy. Cell Rep..

[155] Kyosseva S.V. (2016). Targeting MAPK Signaling in Age-Related Macular Degeneration. Ophthalmol Eye Dis..

[156] Pons M., Cousins S.W., Alcazar O., Striker G.E., Marin-Castaño M.E. (2011). Angiotensin II–Induced MMP-2 Activity and MMP-14 and Basigin Protein Expression Are Mediated via the Angiotensin II Receptor Type 1–Mitogen-Activated Protein Kinase 1 Pathway in Retinal Pigment Epithelium: Implications for Age-Related Macular Degeneration. Am. J. Pathol..

[157] Zou W., Luo S., Zhang Z., Cheng L., Huang X., Ding N., Pan Y., Wu Z. (2021). ASK1/p38-mediated NLRP3 inflammasome signaling pathway contributes to aberrant retinal angiogenesis in diabetic retinopathy. Int. J. Mol. Med..

[158] Tang L., Zhang C., Yang Q., Xie H., Liu D., Tian H., Lu L., Xu J.Y., Li W., Xu G. (2021). Melatonin maintains inner blood-retinal barrier via inhibition of p38/TXNIP/NF-κB pathway in diabetic retinopathy. J. Cell. Physiol..

[159] Lee B.J., Byeon H.E., Cho C.S., Kim Y.H., Kim J.H., Che J.H., Seok S.H., Kwon J.W., Kim J.H., Lee K. (2020). Histamine causes an imbalance between pro-angiogenic and anti-angiogenic factors in the retinal pigment epithelium of diabetic retina via H4 receptor/p38 MAPK axis. BMJ Open Diabetes Res. Care.

[160] Zou W., Zhang Z., Luo S., Cheng L., Huang X., Ding N., Yu J., Pan Y., Wu Z. (2020). p38 promoted retinal micro-angiogenesis through up-regulated RUNX1 expression in diabetic retinopathy. Biosci. Rep..

[161] Du Y., Tang J., Li G., Berti-Mattera L., Lee C.A., Bartkowski D., Gale D., Monahan J., Niesman M.R., Alton G. (2010). Effects of p38 MAPK inhibition on early stages of diabetic retinopathy and sensory nerve function. Investig. Ophthalmol. Vis. Sci..

[162] Dapper J.D., Crish S.D., Pang I.H., Calkins D.J. (2013). Proximal inhibition of p38 MAPK stress signaling prevents distal axonopathy. Neurobiol. Dis..

[163] Seki M., Lipton S.A. (2008). Targeting excitotoxic/free radical signaling pathways for therapeutic intervention in glaucoma. Prog. Brain Res..

[164] Cuenda A., Rousseau S. (2007). p38 MAP-kinases pathway regulation, function and role in human diseases. Biochim. Biophys. Acta.

[165] Schieven G.L. (2009). The p38alpha kinase plays a central role in inflammation. Curr. Top. Med. Chem..

[166] Choy E.H., Panayi G.S. (2001). Cytokine pathways and joint inflammation in rheumatoid arthritis. N. Engl. J. Med..

[167] Stokes D.G., Kremer J.M. (2003). Potential of tumor necrosis factor neutralization strategies in rheumatologic disorders other than rheumatoid arthritis. Semin. Arth. Rheum..

[168] Han J., Jiang Y., Li Z., Kravchenko V.V., Ulevitch R.J. (1997). Activation of the transcription factor MEF2C by the MAP kinase p38 in inflammation. Nature.

[169] Guan Z., Buckman S.Y., Pentland A.P., Templeton D.J., Morrison A.R. (1998). Induction of cyclooxygenase-2 by the activated MEKK1 --> SEK1/MKK4 --> p38 mitogen-activated protein kinase pathway. J. Biol. Chem..

[170] Badger A.M., Roshak A.K., Cook M.N., Newman-Tarr T.M., Swift B.A., Carlson K., Connor J.R., Lee J.C., Gowen M., Lark M.W. (2000). Differential effects of SB 242235, a selective p38 mitogen-activated protein kinase inhibitor, on IL-1 treated bovine and human cartilage/chondrocyte cultures. Osteoarthr. Cartil..

[171] Wiehler S., Cuvelier S.L., Chakrabarti S., Patel K.D. (2004). p38 MAP kinase regulates rapid matrix metalloproteinase-9 release from eosinophils. Biochem. Biophys. Res. Commun..

[172] Da Silva J., Pierrat B., Mary J.L., Lesslauer W. (1997). Blockade of p38 mitogen-activated protein kinase pathway inhibits inducible nitric-oxide synthase expression in mouse astrocytes. J. Biol. Chem..

[173] Koprak S., Staruch M.J., Dumont F.J. (1999). A specific inhibitor of the p38 mitogen activated protein kinase affects differentially the production of various cytokines by activated human T cells: Dependence on CD28 signaling and preferential inhibition of IL-10 production. Cell Immunol..

[174] Ananieva O., Darragh J., Johansen C., Carr J.M., McIlrath J., Park J.M., Wingate A., Monk C.E., Toth R., Santos S.G. (2008). The kinases MSK1 and MSK2 act as negative regulators of Toll-like receptor signaling. Nat. Immunol..

[175] Lang R., Raffi F.A.M. (2019). Dual-Specificity Phosphatases in Immunity and Infection: An Update. Int. J. Mol. Sci..

[176] de la Cruz-Morcillo M.A., García-Cano J., Arias-González L., García-Gil E., Artacho-Cordón F., Ríos-Arrabal S., Valero M.L., Cimas F.J., Serrano-Oviedo L., Villas M.V. (2013). Abrogation of the p38 MAPK α signaling pathway does not promote radioresistance but its activity is required for 5-Fluorouracil-associated radiosensitivity. Cancer Lett..

[177] Lepore Signorile M., Grossi V., Di Franco S., Forte G., Disciglio V., Fasano C., Sanese P., De Marco K., Susca F.C., Mangiapane L.R. (2021). Pharmacological targeting of the novel β-catenin chromatin-associated kinase p38α in colorectal cancer stem cell tumorspheres and organoids. Cell Death Dis..

[178] Pereira L., Igea A., Canovas B., Dolado I., Nebreda A.R. (2013). Inhibition of p38 MAPK sensitizes tumour cells to cisplatin-induced apoptosis mediated by reactive oxygen species and JNK. EMBO Mol. Med..

[179] Roche O., Fernández-Aroca D.M., Arconada-Luque E., García-Flores N., Mellor L.F., Ruiz-Hidalgo M.J., Sánchez-Prieto R. (2020). p38β and Cancer: The Beginning of the Road. Int. J. Mol. Sci..

[180] Sahu V., Nigam L., Agnihotri V., Gupta A., Shekhar S., Subbarao N., Bhaskar S., Dey S. (2019). Diagnostic Significance of p38 Isoforms (p38α, p38β, p38γ, p38δ) in Head and Neck Squamous Cell Carcinoma: Comparative Serum Level Evaluation and Design of Novel Peptide Inhibitor Targeting the Same. Cancer Res. Treat..

[181] Krementsov D.N., Thornton T.M., Teuscher C., Rincon M. (2013). The emerging role of p38 mitogen-activated protein kinase in multiple sclerosis and its models. Mol. Cell. Biol..

[182] Ruano D., Revilla E., Gavilán M.P., Vizuete M.L., Pintado C., Vitorica J., Castaño A. (2006). Role of p38 and inducible nitric oxide synthase in the in vivo dopaminergic cells’ degeneration induced by inflammatory processes after lipopolysaccharide injection. Neuroscience.

[183] Lu G., Kang Y.J., Han J., Herschman H.R., Stefani E., Wang Y. (2006). TAB-1 modulates intracellular localization of p38 MAP kinase and downstream signaling. J. Biol. Chem..

[184] Shi J., Guan J., Jiang B., Brenner D.A., Del Monte F., Ward J.E., Connors L.H., Sawyer D.B., Semigran M.J., Macgillivray T.E. (2010). Amyloidogenic light chains induce cardiomyocyte contractile dysfunction and apoptosis via a non-canonical p38alpha MAPK pathway. Proc. Natl. Acad. Sci. USA.

[185] Mishra S., Guan J., Plovie E., Seldin D.C., Connors L.H., Merlini G., Falk R.H., MacRae C.A., Liao R. (2013). Human amyloidogenic light chain proteins result in cardiac dysfunction, cell death, and early mortality in zebrafish. Am. J. Physiol. Heart Circ. Physiol..

[186] Ohkusu-Tsukada K., Toda M., Udono H., Kawakami Y., Takahashi K. (2010). Targeted inhibition of IL-10-secreting CD25- Treg via p38 MAPK suppression in cancer immunotherapy. Eur. J. Immunol..

[187] Singh R. (2016). Model Predicts That MKP1 and TAB1 Regulate p38α Nuclear Pulse and Its Basal Activity through Positive and Negative Feedback Loops in Response to IL-1. PLoS ONE.

[188] Gupta P., Das P.K., Ukil A. (2015). Antileishmanial effect of 18β-glycyrrhetinic acid is mediated by Toll-like receptor-dependent canonical and noncanonical p38 activation. Antimicrob. Agents Chemother..

[189] Kim L., Del Rio L., Butcher B.A., Mogensen T.H., Paludan S.R., Flavell R.A., Denkers E.Y. (2005). p38 MAPK autophosphorylation drives macrophage IL-12 production during intracellular infection. J. Immunol..

[190] Hallé M., Gomez M.A., Stuible M., Shimizu H., McMaster W.R., Olivier M., Tremblay M.L. (2009). The Leishmania surface protease GP63 cleaves multiple intracellular proteins and actively participates in p38 mitogen-activated protein kinase inactivation. J. Biol. Chem..

[191] Angé M., Castanares-Zapatero D., De Poortere J., Dufeys C., Courtoy G.E., Bouzin C., Quarck R., Bertrand L., Beauloye C., Horman S. (2020). α1AMP-Activated Protein Kinase Protects against Lipopolysaccharide-Induced Endothelial Barrier Disruption via Junctional Reinforcement and Activation of the p38 MAPK/HSP27 Pathway. Int. J. Mol. Sci..

[192] Wang S., Li M., Yin B., Li H., Xiao B., Lǚ K., Huang Z., Li S., He J., Li C. (2017). Shrimp TAB1 interacts with TAK1 and p38 and activates the host innate immune response to bacterial infection. Mol. Immunol..

[193] Makeeva N., Roomans G.M., Welsh N. (2006). Role of TAB1 in nitric oxide-induced p38 activation in insulin-producing cells. Int. J. Biol. Sci..

[194] Makeeva N., Roomans G.M., Myers J.W., Welsh N. (2008). Transforming growth factor-beta-activated protein kinase 1-binding protein (TAB)-1alpha, but not TAB1beta, mediates cytokine-induced p38 mitogen-activated protein kinase phosphorylation and cell death in insulin-producing cells. Endocrinology.

[195] Ohkusu-Tsukada K., Tominaga N., Udono H., Yui K. (2004). Regulation of the maintenance of peripheral T-cell anergy by TAB1-mediated p38 alpha activation. Mol. Cell. Biol..

[196] Ten Hove W., Houben L.A., Raaijmakers J.A.M., Bracke M., Koenderman L. (2007). Differential regulation of TNFα and GM-CSF induced activation of P38 MAPK in neutrophils and eosinophils. Mol. Immunol..

[197] Lanna A., Henson S.M., Escors D., Akbar A.N. (2014). The kinase p38 activated by the metabolic regulator AMPK and scaffold TAB1 drives the senescence of human T cells. Nat. Immunol..

[198] Lanna A., Gomes D.C., Muller-Durovic B., McDonnell T., Escors D., Gilroy D.W., Lee J.H., Karin M., Akbar A.N. (2017). A sestrin-dependent Erk-Jnk-p38 MAPK activation complex inhibits immunity during aging. Nat. Immunol..

[199] Richardson L.S., Taylor R.N., Menon R. (2020). Reversible EMT and MET mediate amnion remodeling during pregnancy and labor. Sci. Signal..

[200] Kang Y.J., Seit-Nebi A., Davis R.J., Han J. (2006). Multiple activation mechanisms of p38alpha mitogen-activated protein kinase. J. Biol. Chem..

[201] Kim S.I., Kwak J.H., Zachariah M., He Y., Wang L., Choi M.E. (2007). TGF-β-activated kinase 1 and TAK1-binding protein 1 cooperate to mediate TGF-β1-induced MKK3-p38 MAPK activation and stimulation of type I collagen. Am. J. Physiol. Ren. Physiol..

[202] Ge B., Xiong X., Jing Q., Mosley J.L., Filose A., Bian D., Huang S., Han J. (2003). TAB1β (Transforming Growth Factor-β-activated Protein Kinase 1-binding Protein 1β), a Novel Splicing Variant of TAB1 That Interacts with p38α but Not TAK1. J. Biol. Chem..

[203] Xin F., Wu J. (2013). Crystal structure of the p38α MAP kinase in complex with a docking peptide from TAB1. Sci. China Life Sci..

[204] Salvador J.M., Mittelstadt P.R., Belova G.I., Fornace A.J., Ashwell J.D. (2005). The autoimmune suppressor Gadd45α inhibits the T cell alternative p38 activation pathway. Nat. Immunol..

[205] Dorn T., Kuhn U., Bungartz G., Stiller S., Bauer M., Ellwart J., Peters T., Scharffetter-Kochanek K., Semmrich M., Laschinger M. (2007). RhoH is important for positive thymocyte selection and T-cell receptor signaling. Blood.

[206] Round J.L., Humphries L.A., Tomassian T., Mittelstadt P., Zhang M., Miceli M.C. (2007). Scaffold protein Dlgh1 coordinates alternative p38 kinase activation, directing T cell receptor signals toward NFAT but not NF-kappaB transcription factors. Nat. Immunol..

[207] Liang Y., Yi P., Wang X., Zhang B., Jie Z., Soong L., Sun J. (2020). Retinoic Acid Modulates Hyperactive T Cell Responses and Protects Vitamin A–Deficient Mice against Persistent Lymphocytic Choriomeningitis Virus Infection. J. Immunol..

[208] Hirata S., Fukamachi T., Sakano H., Tarora A., Saito H., Kobayashi H. (2008). Extracellular acidic environments induce phosphorylation of ZAP-70 in Jurkat T cells. Immunol. Lett..

[209] Giardino Torchia M.L., Dutta D., Mittelstadt P.R., Guha J., Gaida M.M., Fish K., Barr V.A., Akpan I.O., Samelson L.E., Tagad H.D. (2018). Intensity and duration of TCR signaling is limited by p38 phosphorylation of ZAP-70(T293) and destabilization of the signalosome. Proc. Natl. Acad. Sci. USA.

[210] Jun J.E., Kulhanek K.R., Chen H., Chakraborty A., Roose J.P. (2019). Alternative ZAP70-p38 signals prime a classical p38 pathway through LAT and SOS to support regulatory T cell differentiation. Sci. Signal..

[211] Liu J., Guo K., Hu L., Luo T., Ma Y., Zhang Y., Lai W., Guo Z. (2019). ZAP70 deficiency promotes reverse cholesterol transport through MAPK/ERK pathway in Jurkat cell. Mol. Immunol..

